# Annurca Apple Oleolite as Functional Ingredient for the Formulation of Cosmetics with Skin-Antiaging Activity

**DOI:** 10.3390/ijms25031677

**Published:** 2024-01-30

**Authors:** Ritamaria Di Lorenzo, Maria Maisto, Lucia Ricci, Vincenzo Piccolo, Adua Marzocchi, Giovanni Greco, Gian Carlo Tenore, Sonia Laneri

**Affiliations:** 1 RD Cosmetics, Department of Pharmacy, University of Naples Federico II, Via Domenico Montesano 49, 80131 Naples, Italy; ritamaria.dilorenzo@unina.it (R.D.L.); lucia.ricci@unina.it (L.R.); givanni.greco@unina.it (G.G.); sonia.laneri@unina.it (S.L.); 2 ChimNutra Labs, Department of Pharmacy, University of Naples Federico II, Via Domenico Montesano 49, 80131 Naples, Italy; vincenzo.piccolo3@unina.it (V.P.); adua.marzocchi@unina.it (A.M.); giancarlo.tenore@unina.it (G.C.T.)

**Keywords:** Annurca apple, ursolic acid, oleolite, antiaging, wrinkles, cosmeceuticals, topical formulation, elastase inhibition

## Abstract

The identification of natural remedies for the management of the skin aging process is an increasingly growing issue. In this context, ursolic acid (UA), a ubiquitous molecule, mainly contained in Annurca apple (AA) fruit, has demonstrated valuable cosmetic potential. To this end, in the current study, the AA oleolite (AAO, extract in sunflower oil containing 784.40 ± 7.579 µg/mL of UA) was evaluated to inhibit porcine elastase enzymatic reactions through a validated spectrophotometric method. AAO has shown a valuable capacity to contrast the elastase enzyme with a calculated IC_50_ of 212.76 mg/mL, in comparison to UA (IC_50_ of 135.24 μg/mL) pure molecules and quercetin (IC_50_ of 72.47 μg/mL) which are used as positive controls. In this context and in view of the valuable antioxidant potential of AAO, its topical formulation with 2.5% (w/w) AAO was tested in a placebo-controlled, double-blind, two-arm clinical study on 40 volunteers. Our results indicated that after 28 days of treatment, a significant reduction of the nasolabial fold (−7.2 vs. baseline T0, *p* < 0.001) and forehead wrinkles (−5.3 vs. baseline T0, *p* < 0.001) were registered in combination with a valuable improvement of the viscoelastic skin parameters, where skin pliability/firmness (R_0_) and gross elasticity (R_2_) were significantly ameliorated (−13% vs. baseline T0, *p* < 0.001 for R_0_ and +12% vs. baseline T0, *p* < 0.001 for R_2_). Finally, considering the positive correlation between skin elasticity and hydration, the skin moisture was evaluated through the estimation of Trans epidermal water loss (TEWL) and skin conductance.

## 1. Introduction

Skin aging is a complex biological phenomenon deriving from the combination of both intrinsic and extrinsic factors. Specifically, intrinsic aging is a complex and inevitable process depending on several processes, such as cellular metabolism, oxidative stress, the hormonal cycle, genetics, and physiological timing. Extrinsic aging is mainly related to repetitive and prolonged skin exposure to dangerous factors, such as environmental pollution, cigarette smoking, alcohol consumption, use of harsh cosmetics, and principally, sunlight irradiation (photoaging) [1]. This last factor is associated with an increased oxidative stress in the cellular compartments that, in turn, triggers the expression and accumulation of matrix metalloproteinases (MMPs) [2]. These enzymes play a pivotal role in skin tissue degradation by breaking down several structural proteins, including elastin, collagen, and fibronectin [3]. Among all the different kinds of MMPs, skin elastase, also known as MMP-12 [4], is the enzyme capable of degrading elastin [5], thus leading to severe elastosis, dehydration, and wrinkling. Physiologically, elastase plays a pivotal role in tissue remodeling, wound healing, and migration of epithelial cells and macrophages [6]. Since the activity of MMPs increases significantly with age [2], its regulation may be useful in counteracting skin aging [7].

Nowadays, academic research and the cosmetic industry pay considerable attention to natural bioactive compounds to develop innovative and sustainable cosmetic formulations. Several food- and plant-derived molecules have shown remarkable cosmetic potential [8,9,10]. Oleuropein, a polyphenol extracted from olive leaves and olive oil, was found to exert protective and lenitive activity when topically applied on the skin of volunteers before UVB exposure [11]. Quercetin exhibited relevant anti-inflammatory and anti-acne properties both in vitro [12] and in clinical studies [13]. Moreover, several plant-derived antioxidants, due to their important reducing activity, are largely used for the formulation of lotions and creams [8].

The reutilization and valorization of food waste products as a source of bioactive molecules for the formulation of nutraceuticals and cosmeceuticals is an environmentally sustainable increasing trend worldwide. Considering that the global production of food loss and waste represents 17% of the total production process, with an estimated annual cost of USD 2.6 trillion, food waste reutilization has found a great appeal in the cosmeceutical industry [14].

A recent article reported that the topical application of pomegranate seed oil (the seeds are a massive waste product formed during pomegranate processing) is particularly effective in contrasting UV-induced stress and damage in animals and in vitro models [15], as well as chitin and pectin isolated from different types of food waste being applied as a functional ingredient for the preparation of beauty masks [16]. In this sense, in a recent paper it was reported that purified bromelain obtained from pineapple peel, stem, and crown was used to prepare a facewash formulation to contrast skin infections [17]. In line with this trend, Costa and colleagues have formulated microemulsions and macroemulsions for topical application incorporated with lycopene-enriched extracts from tomato waste, in order to investigate their potential skin protective effects [18].

Ursolic acid (UA), a ubiquitous triterpenic acid, with a well-described antioxidant activity [19], is widely used as a functional ingredient for the preparation of topical formulations with various applications, including acne control [20], prevention of skin roughness [21], and modulation of epidermal permeability [22]. UA has gained particular attention in the cosmeceutical field due to its potent anti-aging activity, primarily related to its ability to modulate the activity of the elastase enzyme [23]. The most important food sources of UA are generally apple fruit [24], and the Annurca apple (AA) has been shown to be one of the richest ones among the many apple varieties [25]. AA is the only apple cultivar native to southern Italy that the European Council has registered as a Protected Geographical Indication (PGI) product [Commission Regulation (EC) No.417/2006)] [26]. While the nutraceutical potential of the polyphenolic portion of AA has been largely described for its beneficial effects on plasma cholesterol levels [27,28,29], hair tropism [30], and antidiabetic effects [29], without any toxic effects, the nutraceutical and cosmeceutical potential of AA triterpenic fraction is not as well known.

The optimization of the UA extraction process in sunflower oil as a biocompatible solvent from lyophilized AA was described in detail in our recent article. The obtained oleolite with a UA concentration of 750 μg/mL demonstrated desirable skin permeation properties suitable for potential cosmeceutical applications. In the current study, to explore such potential of the AA oleolite (AAO), its activity on elastase enzyme was investigated through a validated enzymatic spectrophotometric in vitro assay. The anti-elastase activity exhibited by AAO prompted us to undertake a cosmetic double-blind clinical trial to evaluate the AAO-based topical formulation efficacy on 40 subjects with various skin aging-related parameters, such as elasticity, firmness, and roughness, after ensuring its skin tolerability.

## 2. Results

### 2.1. Validation of Elastase Inhibition Assay

**Figure 1:**
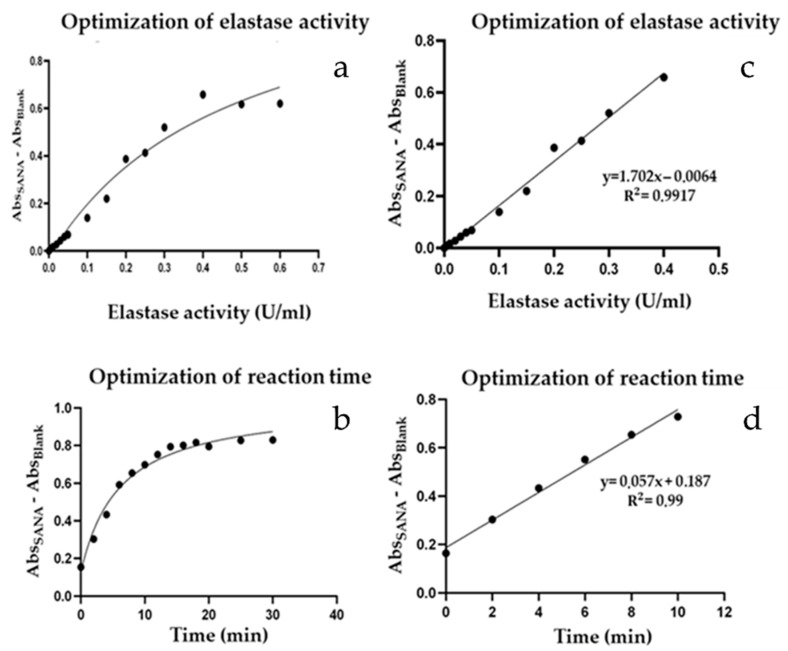
Optimization of elastase inhibition assay conditions. (**a**) Optimization of elastase activity; (**b**) optimization of reaction time; (**c**) linear kinetics of enzymatic reaction on the base of elastase activity; and (**d**) linear kinetics of enzymatic reaction on the base of reaction time. Values represent the mean ± of three replicates analyzed.

**Figure 2:**
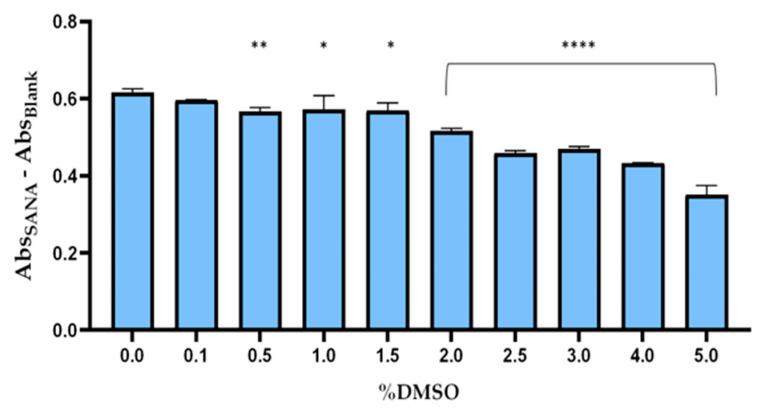
Optimization of %DMSO in enzymatic assay. * *p-*values < 0.05, ** *p-*values < 0.01; **** *p-*values < 0.001.

Generally, the evaluation of elastase inhibition was performed by a spectrophotometric method based on the detection of *p*-aniline at 400 nm [31]. Practically, this assay was based on the elastase enzymatic hydrolysis of succinyl-(Ala)-3-p-nitroanilide (SANA), in p-nitroanilide which was spectrophotometrically monitored. In order to validate the optimal reaction conditions for the enzyme reaction, the following parameters were optimized: elastase enzymatic activity (0.005–0.6 U/mL) (Figure 1a,c), incubation time (0–30 min) (Figure 1b,d), and DMSO concentrations (0–5%) (Figure 2). After determining these conditions, the reaction course was also examined to establish the values of the specific parameters whose enzymatic reaction exhibited linear kinetics (Figure 1c,d). Specifically, our results indicated that the minimum value for each factor investigated that resulted in the maximum *p*-nitroanilide production was as follows: elastase concentration of 0.4 U/mL (Figure 1c), the reaction time of 10 min (Figure 1d), and DMSO 1.5% (Figure 2). Thus, these conditions were combined to set the experimental protocol of this spectrophotometric assay.

### 2.2. Elastase Inhibition Assay

**Figure 3:**
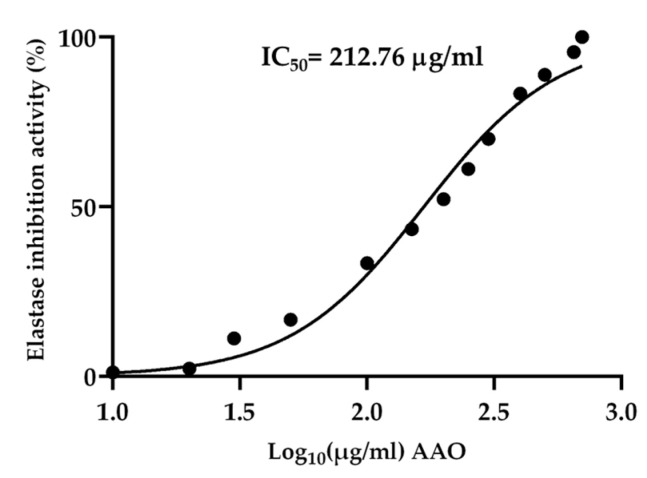
Inhibition of elastase enzyme (%) by AAO calculated as IC_50_. Values represent the mean ± of three replicates analyzed.

**Figure 4:**
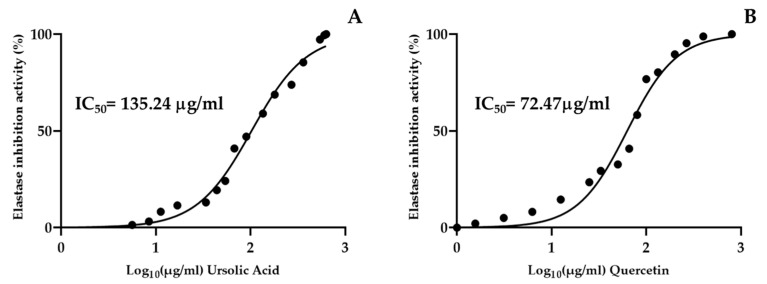
Inhibition of elastase enzyme (%) by ursolic acid (**A**) and quercetin (**B**) calculated as IC_50_. Values represent the mean ± of three replicates analyzed.

Elastase plays a pivotal role in the skin aging process, thus inhibiting this enzyme represents a possible preventive approach for the management of skin-elasticity loss and its related wrinkles. To evaluate the capacity of AAO as a potential anti-aging functional ingredient in cosmeceutical formulation, the AAO in vitro inhibitory activity on elastase was investigated. As reported in Figure 3, the AAO has shown a remarkable ability to inhibit elastase activity in a concentration-dependent manner, with a calculated IC_50_ of 212.76 μg/mL. To corroborate the hypothesis that UA was the main molecule responsible for elastase inhibition, the pure UA was assayed, resulting in IC_50_ 135.24 µg/mL (Figure 4B), which is approximately, two-fold more active than the AAO. In addition, as a positive control, quercetin and flavanol are largely produced in apples, with strong chelating properties for copper at the active site of the elastase enzyme [32]. Predictably, the quercetin IC_50_ (72.49 µg/mL) was the lowest calculated value in the current study (Figure 4A).

### 2.3. Skin Visco-Elasticity

To confirm the promising in vitro results on the enzymatic activity of elastase, the ability of the formulated AAO to increase the viscoelastic properties of the skin in enrolled subjects was investigated. In particular, the cyclic suction/release method describes the viscoelastic behavior of the skin well [33,34]. The controlled air depressor system and optical displacement measurement of Cutometer MPA 580 (Courage + Khazaka electronic GmbH, Köln, Germany) allowed in vivo evaluation of skin biomechanical properties, by monitoring R_0_ and R_2_ parameters, indicating, respectively, the skin pliability/firmness (mm) and the gross elasticity (%). Through this in vivo assay, the in vitro demonstrated AAO inhibitory action on elastase can be directly related to an overall improvement of the skin biomechanics. As reported in Table 1, the AAO revealed a noticeable skin elasticizing and firming activity in a time-dependent manner, when used at a topical concentration of 2.5%_w/w_. Gross elasticity (R_2_) increased by 14.0% and 28.0% after 14 (D_14_) and 28 (D_28_) days of treatment, and analogously tonicity was improved by AAO topical application with an average percentage recorded variation of −9.0% and −13.0%, respectively after 14 and 28 days of treatment. The placebo formula did not show the same efficacy in improving skin biomechanics.

### 2.4. Skin Hydration and Film-Repairing Activity

Relationships between skin visco-elasticity and hydration have been widely discussed [35], thus in the current study, the ability of AAO topical formulation to ameliorate the skin hydration condition was investigated. To this end, the skin conductance and the Trans epidermal water loss (TEWL) were measured at different time points on enrolled subjects. Conductance measures skin hydration non-invasively by evaluating its electrical properties, as water has a higher dielectric constant than most other substances. Skin conductivity results indicated a higher water content in the stratum corneum (T_1h_: 17.8%; T_24h_: 17.2%), since the first application of AOO, which was further confirmed at the end of the test (D_14_: 20.0%; D_28_: 25.0%). Similarly, TEWL measurements suggested a recovery action on the unpaired skin barrier, both in the short- (T_1h_: −13.2%; T_24h_: −13.4%) and long-term treatment (D_14_: −17.0%; D_28_: −23.0%). AAO ameliorated skin barrier conditions and boosted hydration with statistically significant differences with the vehicle formulation, which showed no significant effect on these skin conditions (Table 2).

**Figure 5:**
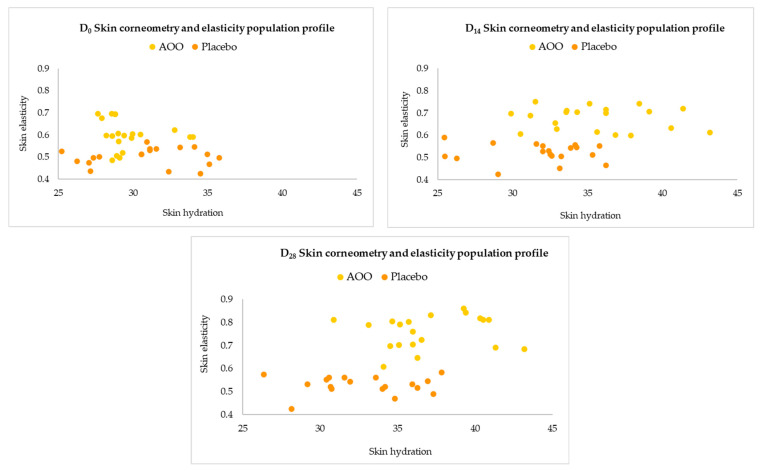
Distribution profile of skin hydration and elasticity values of the population under treatment with topical 2.5%_w/w_ AOO and Placebo.

Furthermore, in order to confirm the previously described relationship between skin elasticity and hydration, a correlation study between the obtained experimental skin hydration and elasticity values at the different time points (D_0_, D_14_, and D_28_) (Figure 5) was performed. Our results indicate that treatment with AAO disrupts the basal condition, leading to a population with higher hydration and elasticity values. Specifically, the study found that AAO treatment caused a perturbation in the skin’s natural state, increasing hydration and elasticity values. Moreover, during AAO treatment, the population underwent a perturbation that resulted in a tendency of linearity between hydration and elasticity, indicating that the oleolite acts by stimulating the skin on both aspects. This is in contrast to the placebo group, which does not experience any disruption in its distribution profile; therefore, this suggests a potential use for AAO as a cosmetic ingredient in skincare products aimed at improving skin hydration and elasticity.

### 2.5. Wrinkles and Fine Lines

**Figure 6:**
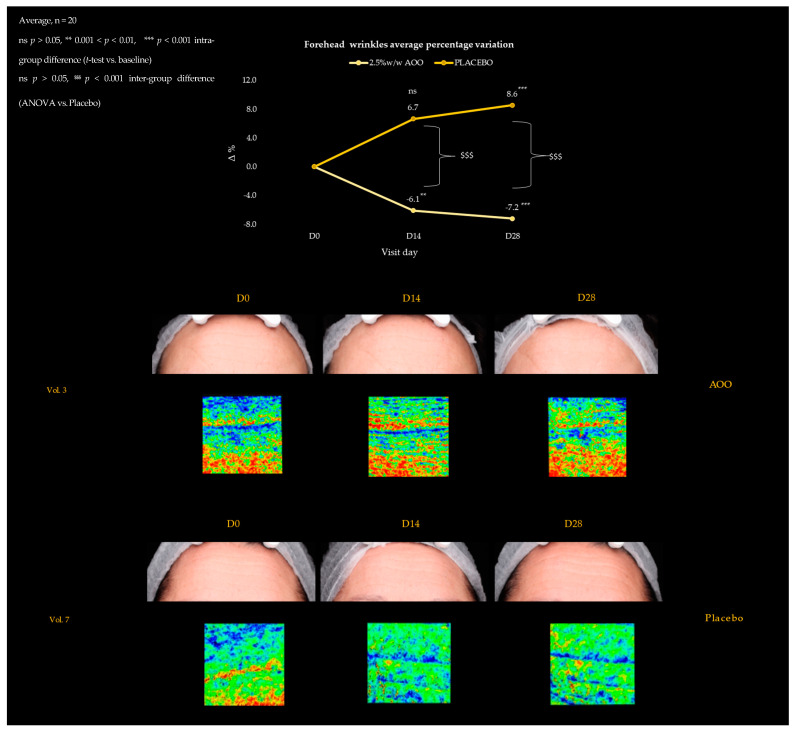
Two-dimensional and three-dimensional forehead wrinkles over 28-day treatment with 2.5%_w/w_ AAO vs. Placebo. The three-dimensional replica showed in blue, skin furrows, while hollows in red. The AOO group reduced the blue replica area demonstrating the smoothing ability of the oleolite in treating expression lines.

**Figure 7:**
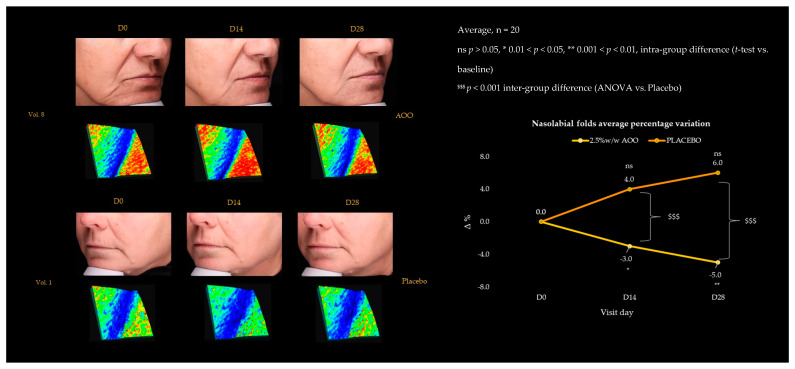
Two-dimensional and three-dimensional nasolabial folds over 28-day treatment with 2.5%_w/w_ AAO vs. Placebo. The three-dimensional replica showed in blue, skin furrows, while hollows in red. The AAO group reduced the blue replica area indicating the smoothing ability of the oleolite in treating gravitational lines.

The aging process alters both the structure and mechanical properties of the skin, mainly through changes in the collagen and elastic fibers of the dermis [36]. As a result, the skin gets more wrinkles [37]. Microscopically, the fine mesh of the skin surface declines, and each wrinkle becomes prominent, as its width and height grow with age [38]. Thus, the demonstrated inhibitory action of AAO on elastase enzyme can, with reasonable predictivity, have a pivotal role in the phenotypic manifestations of skin ageing, like wrinkles and fine lines appearance. As evidence, our study demonstrated that AAO treatment decreased the most evident facial wrinkles. Specifically, forehead and frown lines were detected through VISIA 7th (Canfield Scientific Inc., Parsippany, NJ, USA) in the forehead area, which showed a statistically significant decrease over time by −6.1 and −7.2% at D_14_ and D_28_ vs. baseline (Figure 6). Likewise, nasolabial folds reduced their intensity by −3.0 and −5.0% during the 28-day treatment period with topical AAO (Figure 7), contrary to what is observed for the placebo-treated group. These results demonstrated the AAO validity in the treatment of the most typical skin ageing disorders.

### 2.6. Skin Tolerability

**Figure 8:**
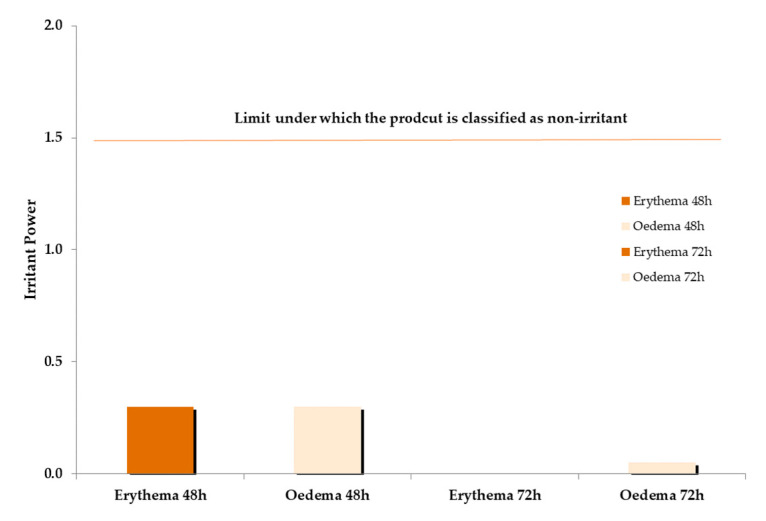
Average erythema and oedema index value at 48 and 72 h.

Before establishing the potential anti-aging activity of the studied formulation, its skin tolerability was ensured. To this end, the cosmetic product Irritant Power (IP) of AAO was evaluated. Specifically, the AAO potential skin irritant activity was evaluated through an occlusive 48 h patch test, which revealed that AAO used at 2.5%_w/w_ in topical formulations is not irritating. The recorded IP at 48 h (IP48) and at 72 h (IP72), calculated on 20 subjects was lower than 1.5, the limit under which topical products are generally classified as a non-irritant [39] (Figure 8).

## 3. Discussion

Elastase, a protease enzyme present in the dermal layers, effectively degrades skin elastin leading to alterations in elasticity. As a consequence, the reduced elastin skin content was positively correlated to skin age-related changes, including skin wrinkles, roughness, and loss of skin elasticity [25]. Thus, the incorporation of elastase inhibitors in cosmetic formulations could support skin aging by maintaining skin elasticity and preventing sagging and laxity. Recently, there has been great attention on the individuation of natural bioactive molecules with potential anti-elastase activity. Specifically, it was well-established that UA was a natural compound with multi-target cosmeceutical potential. Chemically, UA was a triterpenic acid, mainly contained in apple peel [40]. The main limitation of its nutraceutical or cosmeceutical application was related to its high lipophilicity, which makes necessary the use of apolar, but not biocompatible, solvents for UA recovery from several food sources. Consequently, to overcome this practical problem, in our previous publication, the UA extraction from AA in sunflower oil as a biocompatible and lipophilic solvent was optimized in order to reach a UA concentration of 762 μg/mL in Annurca apple oleolite, AAO [9]. Thus, in line with these considerations, in order to explore the AAO cosmeceutical application, we evaluated the inhibitory potential of the formulated AAO on elastase activity using an optimized enzymatic reaction assay. Our results show that the formulated AAO was able to inhibit the elastase activity with a calculated IC_50_ of 286.42 mg/mL. This promising result could be mainly due to AAO-relevant UA content, which has been shown to be a potent elastase inhibitor agent, with a calculated IC_50_ of 26.8 μg/mL (0.026 μM). In this regard, from the molecular point of view it was described that UA was able to contrast the activity of elastase enzyme by binding to the enzyme’s subsites S3–S5 [41]. Specifically, it was reported that the main structural characteristic of the elastase inhibitor is the presence of hydroxyl groups. This explains why the triterpene glycoside has no elastase activity: the bulky hydrophilic glycosyl residues hinder the interaction of the constitutive aglycon with the substrate-binding site of the enzyme [41]. Therefore, although UA is the major triterpenic compound detected in the prepared oleolite, the inhibitory activity of AAO towards elastase may be also potentiated by the activity of apolar polyphenols identified in AAO which are phloridzin (0.15 ± 0.010 mg/mL), phloretin (0.07 ± 0.01 mg/mL), rutin (0.76 ± 0.001 mg/mL) and quercetin-3-*O*-glucoside (0.71 ± 0.007 mg/mL) [9]. Regarding the anti-elastase effect of polyphenols, quercetin generally proven to be the most active polyphenol, which is why quercetin was used as a positive control in an elastase in vitro inhibition assay. This observation was confirmed by our data that reported quercetin IC_50_ of 74.72 μg/mL, a value perfectly in line with the finding of another author, which described quercetin IC_50_ for porcine elastase inhibition ranging from 60 to 70 μg/mL [32]. Considering that the main AAO polyphenolic components were quercetin derivatives, such as rutin and quercetin-3-*O*-glucoside, it could be assumed that they act synergically with UA in inhibiting elastase activity. However, the valuable activity of both rutin and quercetin-3-*O*-glucoside on elastase enzyme was well-documented [42].

Once established that AAO was able to contrast the elastase activity in the in vitro assay, to confirm such promising antiaging activity, an instrumental clinical trial using AAO-based topical formulation (2.5% (w/w)) was performed on healthy human subjects, after ensuring its skin tolerability through a patch test. According to the collected data, the formulated AAO-based product is safe and not an irritant for topical applications. Additionally, in accordance with the in vitro results, the in vivo evidence indicates a relevant antiaging effect in terms of the reduction of skin wrinkles at the nasolabial and T area levels in treated subjects. Specifically, our results indicated a statistically significant reduction after 28 days of treatment of nasolabial fold and forehead wrinkles vs. T0 parameters of −5.0% and −7.2%, respectively.

These results are supported by those described by others in different in vitro cellular models, who reported that UA-based topical formulation has shown a relevant capacity to induce collagen content in cultured normal human dermal fibroblasts [43].

Additionally, the improvement of the skin visco-elastic parameters R_0_ e R_2_ were evaluated in enrolled subjects. Actually, according to the present scientific literature, there is no evidence related to the in vivo UA modulation of such visco-elastic factors, while other cosmeceutical formulations have shown a valuable ability in the management of these parameters. In this context, a saffron-based topical formulation has shown a valuable amelioration of elastic skin parameters, such as net elasticity (R_5_), and gross elasticity (R2) after 12 weeks of treatment in 20 healthy patients [44]. Finally, considering the well-established relationship between skin elasticity and hydration, the skin moisture parameters were investigated. Our results indicate that after 28 days of treatment, a valuable increase of dermal hydration in terms of skin conductance (+25% vs. T0, *p* < 0.001) was observed in the AAO group, combined with a significative reduction of skin TEWL (−23% vs. T0, *p* < 0.001). The ability of UA-based cosmeceutical formulation to modulate skin hydration parameters was also previously evaluated in postmenopausal volunteers, where after treatment, a valuable reduction of transepidermal water loss improved skin moisturization, reduced scaliness, and improved skin elasticity [45]. In addition, our results were supported by in vitro results, which describe the positive effect of triterpenic acids, especially UA and oleanolic acid, on the epidermal permeability barrier and therefore on skin moisture, indicating a valuable improvement in skin moisture after application of UA [46]. Additionally in our study, a positive correlation between gross elasticity (R_2_) and hydration was calculated (Figure 5). This finding confirms the previous theory proposed by Debelle et al. [47], who insisted that only the water-swollen elastic fibers have an elastic recovery ability that improves the firmness of the skin.

In any case, it is not negligible that the antioxidant activity of the oleolite, previously evaluated [9], could play a pivotal role in skin aging protective effects. AAO has shown valuable antiradical (DPPH and ABTS test) and ferric-reducing activity (FRAP-test), with a calculated IC_50_ of 14.63, 5.90, and 21.72 ± 0.68 µmol (Trolox equivalent (TE)/g of AAO), respectively. Considering that oxidative stress plays a crucial role in the appearance of the clinical manifestation of skin aging, accelerating the molecular mechanisms that underline this natural phenomenon [2], the AAO antioxidant potential could have a positive impact on the skin condition. In conclusion, the current study paves the way for possible new applications of this oleolite as a valuable cosmeceutical ingredient, hitherto only predicted, and perfectly in line with the most current trend pursued by cosmetic industries to employ sustainable and upcycled active ingredients [48].

## 4. Materials and Methods

### 4.1. Reagents

All chemicals, reagents, and standards used were analytical or LC-MS grade reagents. The water was treated in a Milli-Q water purification system (Millipore, Bedford, Burlington, MA, USA) before use. Sunflower oil was purchased in a local market. Ursolic acid (purity ≥ 98.5% HPLC), phloridzin (purity ≥ 99% HPLC) were purchased from Sigma-Aldrich (Milan, Italy). For the o/w topical formulation, cosmetic-grade ingredients were used, such as Glyceryl-Stearate and PEG-100 Stearate, Caprylic/Capric Triglycerides, Cetearyl Alcohol, Cetyl Ricinoleate, Sodium Gluconate, and Carbomer, Phenoxyethanol (and) Ethylhexylglycerin. All listed excipients were purchased from ACEF Spa (Fiorenzuola D’arda, Italy).

### 4.2. Oleolite Preparation and Extraction of Triterpenic Fraction

Annurca apple fruits (Malus pumila Miller cv Annurca) (about 100 g each) were collected in October 2022, Valle di Maddaloni (Caserta, Italy), when the fruits had just been harvested (green peel). The fruits were reddened for about 30 days following the typical treatment [26]. After this time, the apples were washed and sliced for freeze-drying. The oleolite preparation was performed according to the conditions optimized in our previously published article [9] to achieve the maximum UA concentration of 784.40 ± 7.579 (mg/mL). Specifically, the ratio of freeze-dried AA to sunflower oil was 1:4, and the mixture was stirred at 68.85 °C for 63 h. Then, the AAO was subjected to a specific ethyl acetate extraction protocol for the isolation of the triterpenoid acids, according to our published protocols. This extract was used to verify the effect on the elastase enzyme. All the results observed are expressed in terms of mg of AAO [9]. Then, the AAO was subjected to two different extraction protocols for the isolation of the triterpenoid acids and polyphenols fractions, according to our published protocols. The two extractions were performed with ethyl acetate and hydroalcoholic mixtures, respectively. The fractions obtained were first dried using a rotary evaporator, when the solvents used were completely removed, the residues obtained were suspended together in water, frozen at −80° and freeze-dried (for 24 h at −69 °C at 0.096 mbar). The resulting powder was ground (IKA A11 analytical mill) to obtain a homogeneous powder representative of the triterpenic acids and polyphenol fractions. The chemical composition of the prepared extract was assessed by HPLC-DAD analysis, leading to the identification and quantification of 6 different polyphenolic compounds: Rutin (0.76 ± 0.001); Quercetin-3-*O*-glucoside (0.71 ± 0.007); Kaempherol-3-*O*-rhamnoside (0.15 ± 0.010); Apigenin-7-*O*-glucoside (0.0081 ± 0.001); Phloridzin (0.1 ± 0.010); and Phloretin (0.07 ± 0.01) μg/mL of AAO and UA at the concentration of 750 μg/mL. All the results observed are expressed in terms of mg of AAO.

### 4.3. Elastase Inhibition Assay

The elastase inhibition assay was performed according to the previously reported spectrophotometric method [31], with slight modifications. Practically, this method was based on the enzymatic activity of elastase, which is involved in an oxidation reaction of the peptide linkage of the substrate succinyl-(Ala)-3-p-nitroanilide (SANA) to *p*-nitroanilide, a cleavage product which is monitored at a wavelength of 410 nm. An amount of 10 µL of different concentrations of the samples (AAO, UA, quercetin) and 100 µL of 0.5 mM of N-succinyl-(Ala)-3-p-nitroanilide were added to a 96-well microplate. After an incubation time of 10 min, 10 µL of elastase from porcine pancreas (0.4 U/mL) was added, mixed well, incubated at room temperature for 10 min, and finally the absorbance was determined at 410 nm using a microplate reader. An amount of 0.5 mM of N-succinyl-(Ala)-3-p-nitroanilide and the enzyme elastase (0.4 U/mL) were prepared in a 0.1 M Tris-HCl buffer at pH 8.0. The analyzed standards and the extracts were dissolved in 100% *v*/*v* DMSO and diluted with 0.1 M Tris–HCl buffer at pH 8.0 to obtain a final concentration of 1.5% *v*/*v* DMSO in the enzymatic mixture. The performance of the assay was verified using quercetin as a reference and UA as a principal bioactive triterpenic acid in AAO, under the same assay conditions. The inhibitory effect was expressed as the concentration required to inhibit elastase activity by 50% (IC50). Generally, the capacity to inhibit an enzymatic reaction was expressed as a % of the inhibition [49], calculated as Inhibition (%) = (1 − B/A) × 100, where A is the enzyme activity without the sample and B is the activity in the presence of the sample.

### 4.4. AAO-Based Topical Formulation Preparation

Cosmetic o/w emulsions were prepared with cosmetic-grade ingredients. First, phase A containing oily constituents, such as Glyceryl-Stearate and PEG-100 Stearate (4.00%_w/w_), Caprylic/Capric Triglycerides (10.00%_w/w_), Cetearyl Alcohol (0.50%_w/w_), and Cetyl Ricinoleate (2.00%_w/w_) were mixed using a magnetic stirrer at 200 ± 25 rpm and heated up to 70 ± 5 °C.

Once melting occurred, phase A was mixed with phase B (aqueous phase), comprising deionized water (79.55%_w/w_), Sodium Gluconate (0.10%_w/w_), and Carbomer (0.35%_w/w_), previously heated at the same temperature of phase A. Phases mixing occurred using the mechanical stirrer Silverson L5T Laboratory Mixer (SBL, Shanghai, China), at 4500–5000 rpm until the 50 ± 2 °C temperature was reached, then at 2500–3000 rpm until the mix became supple and homogenous. Once 37 ± 2 °C was reached, the final phase (phase C) containing the preservative system, such as water solution of Phenoxyethanol (and) Ethylhexylglycerin (0.90%_w/w_), and the active ingredient AAO (2.50%_w/w_), was added to the mix. For the clinical trial, two batches of the topical formulation were prepared, such as the placebo and active batch. The active batch was enriched with the active ingredient AAO (2.50%_w/w_) in phase C, before neutralization with NaOH 2N sol. which occurred once the emulsions were cool.

#### Quality Control and Potting of the Cosmetic Formulations

After 24 h, viscosity (18.750–19.633 mPa; 22 °C, spindle 64, 20 rpm) was measured using the Brookfield DV-E viscosimeter (AMETEK Inc., Berwyn, PA, USA), and the pH (5.6 ± 0.3) was checked with the GPL20 pH-Meter (Crison Instruments S.A., Barcelona, Spain). The placebo emulsions were prepared analogously and contained all the listed ingredients, except for the AA oleolite. Once ready, cosmetic creams were placed in anonymous 30 mL jars, by a blind technician, in blind conditions.

### 4.5. Clinical Study

This randomized, double-blind, efficacy study was performed at the RD Cosmetics laboratory of the University of Naples Federico II—Pharmacy Department, which has a quality management system for cosmetic clinical trials certified by RINA S.p.A (Genova, Italy) according to the UNI EN ISO 9001 standard [50]. The cosmetic clinical trial aimed to assess the skin tolerability and the anti-aging efficacy of the AAO vs. placebo incorporated in a topical formulation. Due to the cosmetic nature of the present study and the involvement of adult healthy human subjects, a submission to the relevant ethics committee was not required. The clinical study was performed after written informed consent (S1-Supplementary Materials) was provided by all subjects. Nevertheless, the study was performed in accordance with the principles embodied by the Declaration of Helsinki [51] and following COLIPA guidelines for the Evaluation of the Efficacy of Cosmetic Products [52]. Good clinical practice was maintained throughout the study.

#### 4.5.1. Study Design and Participants

40 subjects aged 40 to 65 years (included) with relaxed skin tone and dermis thinning were eligible for the study.

Further inclusion criteria were discontinuation of all cosmetic products used to treat aging phenomena from screening up until the final follow-up visit; however, cleansing preparations deemed acceptable by the investigator and make-up were permitted. Exclusion criteria included any dermatological disease, previous use of retinoids, and use of medical aesthetic procedures, such as bio-stimulations, filler or botox within 12 weeks or five half-lives of screening, face radiofrequencies within 8 weeks of screening, or anti-aging/antioxidant oral supplementation within 4 weeks of screening. Further exclusion criteria were the presence of both topical and systemic pathologies or diseases. Characteristics of the study populations are shown in Table 3.

**Figure 9:**
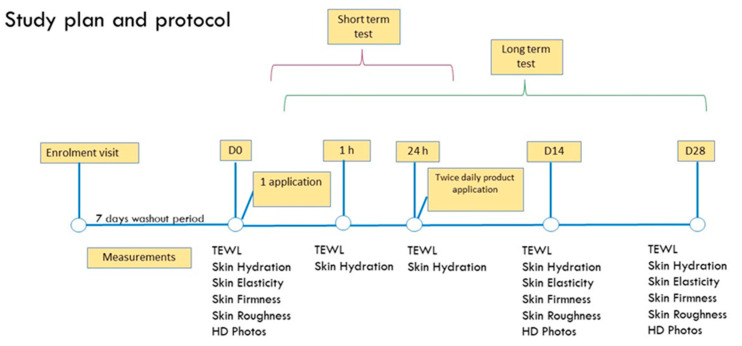
Timeline scheme of the study. D_0_: before test product topical application; 1 h: 1 h after product application; 24 h: 24 h after product application; D_14_: 14 days after topical treatment; and D_28_: 28 days after topical treatment.

After the enrollment visit, a mandatory washout period of 7 days was required before study entry. During the baseline (D_0_) and check-up visits, participants were assessed for the monitored skin parameters and were randomly assigned to a treatment group. They received test samples and were instructed about the way of use. They had to apply about 2 mg of the assigned product on their face twice daily (morning and evening) for 28 days. An overview of the study plan and protocol is illustrated in Figure 9.

The primary outcome of the study was the determination of the skin aging-related parameter improvement (skin elasticity, firmness and roughness) and AOO skin tolerability; the secondary outcomes were transepidermal water loss (TEWL) and corneometry assessment in accordance with the literature findings [17], with a short- and long-term test.

#### 4.5.2. Randomization and Masking

**Figure 10:**
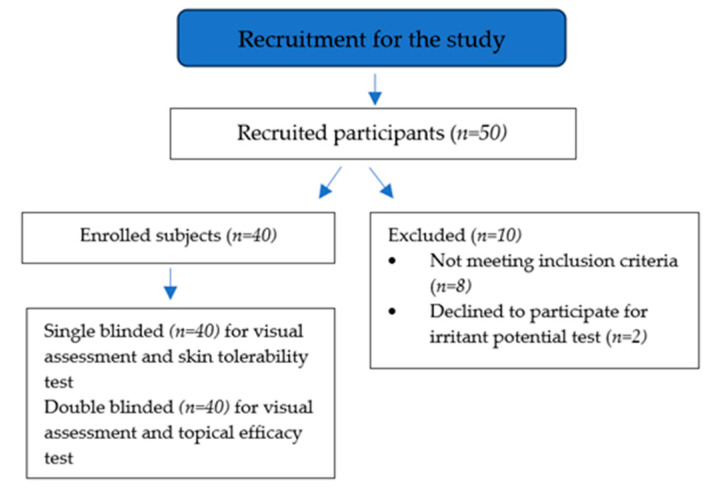
Flow diagram of participant selection and clinical assessments (irritant potential and topical efficacy) performed.

The laboratory staff used a system for the management of study enrollment and ensuring anonymity. The system assigned to the enrolled subjects a patient study number, randomized according to defined parameters drawn on a general panel where volunteers are recorded, and maintained treatment masking. Subjects came from all social categories, and they spontaneously participated in the study. Before admission, they received a clinical skin examination and a detailed cosmetic questionnaire. The system used a configurable buildable algorithm with limits set to patient age (≤40 and >65 years) and skin type (thin dermis and relaxed face). Patients were randomly assigned AAO-based cream (2.5%_w/w_ twice daily) or vehicle cream twice daily (placebo group) in a 1:1 ratio. Patients, and investigators, (except members of the primary endpoint analysis data monitoring teams) remained masked to each patient’s treatment assignment throughout the study. Emergency unmasking was conducted in case of side effects that required the investigator’s knowledge of the patient’s treatment group. At the end of the study, the number of patients who respected the protocol with no significant deviation, which could influence the study results, had to be 20 for each treatment group (Figure 10).

#### 4.5.3. Anti-Aging Efficacy Assessment

Skin firmness and elasticity were assessed after 14 and 28 days (D_14_ and D_28_) of treatment with AOO-based cream or placebo using Cutometer**^®^** Dual MPA 580 (C + K electronic GmbH, Cologne, Germany), while wrinkles and fine lines were determined through evaluation of forehead and nasolabial wrinkles with VISIA 7th (Canfield Scientific Inc., Parsippany, NJ, USA).

#### 4.5.4. Short- and Long-Term Hydration and TEWL

Hydration and TEWL were evaluated after one and repetitive application of the investigated topical formulations using Corneometer**^®^** CM 825 and Tewameter**^®^** TM Hex (C + K electronic GmbH, Cologne, Germany). All parameters were evaluated at baseline (D_0_) after 1 and 24 h by assigned product application (1 h and 24 h, respectively), and on days 14 and 28 (D_14_ and D_28_).

#### 4.5.5. Skin Tolerability Test

In order to ensure the test product tolerability, its potential irritant power was evaluated when it is applied in a single dose to intact human skin with an occlusive patch test for 48 h. The irritancy patch test was conducted on the volar forearm due to its hairlessness, lower sebaceous gland count, and larger skin surface area. This test allowed for the evaluation of the tolerability of the AAO when transmitted in a topical formulation, through the identification and classification of its potential irritant power, as required by EEC Directive 76/768 [52].

The test is conducted using Finn Chambers^®^ AQUA (Bio-Diagnostics Ltd., Worcester, UK) which is a patch delivery system and holding device to place allergens and allergen mixes in contact with the surface of the skin during allergen patch tests. The AAO cream and placebo were displaced directly into different chambers and applied to healthy skin on the upper forearm, which was clean, dry, and free of ointments or lotions. The panel was left in situ for 48 h, then, the panel was removed, and skin reactions were recorded (PI48), and measured again after a further 24 h (PI72). Results of the patch test are measured by morphologic criteria recommended by the International Contact Dermatitis Research Group [53], using a 2 × magnifying lamp for visual scoring using the following grading system: 0 = no evidence of reaction, 1 = minimal erythema, 2 = visible erythema and 3 = erythema and papules. The irritancy power was calculated using the average visual score + standard deviation (SD) for 2.5%_w/w_ AOO formulation and the placebo—irritancy levels for the sample were classified as follows: average clinical score + SD > 1.5 = sample is an irritant; average clinical score + SD ≤ 1.5 and > negative control = sample has low irritancy potential and a mean visual score + SD ≤ negative control = sample is non-irritant. The irritancy limit of 1.5 on a 0–3 scale for the visual score + one SD was used as the irritancy level threshold.

### 4.6. Statistical Analysis

A sample size of 40 randomly assigned patients (approximately 20 patients per group) was determined to provide sufficient statistical power to detect the difference between the AAO cream and placebo. For the primary and secondary endpoints, comparisons between the AAO groups and placebo were conducted using the ANOVA test (inter-groups differences), whereas average percentage variations compared to baseline for each group were controlled using the Student *t*-test (intra-groups differences) at an overall two-sided *p* of 0.05. Statistical analyses were done with SAS software version 9.4. Regarding the in vitro assay, GraphPad Prism software version 9 (San Diego, CA, USA) was used for statistical analysis. The t-test and ANOVA test were used for the comparison of two groups or multiple groups, respectively. The data were shown as mean ± DS. A *p*-value < 0.05 was considered statistically significant and was labeled with *; *p-*values < 0.05, 0.01 or 0.001 were labeled with **, *** or ****, respectively.

## 5. Conclusions

The described results show that AAO (containing 784.40 ± 7.579 µg/mL of UA) is a precious source of natural bioactive compounds, especially of UA, useful for the management of aging-related skin disorders. AAO has shown a valuable ability to inhibit elastase with an estimated IC_50_ of 212.76 mg/mL. This in vitro evidence was corroborated by valuable clinical results, where the treatment of 20 healthy subjects with AAO-based topical formulation (2.5% of AAO (w/w)) led to a noticeable decrease of the nasolabial fold and forehead wrinkles, improvement of visco-elastic parameters, and remarkable amelioration of the skin hydration condition. Thus, in conclusion, the formulated AAO could be considered a potential functional ingredient for the formulation of cosmeceutical products with skin anti-aging activity. Undoubtedly, further investigations are necessary to highlight the potential molecular mechanism of actions responsible for the registered clinical evidence.

## Figures and Tables

**Table 1:** Skin biomechanical properties (R_0_ and R_2_ average value ± SD) before and after treatment with 2.5%_w/w_ AAO or Placebo.

	Visit Day 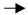 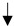 Skin Parameter	Baseline (D_0_) Mean ± SD	Day 14 (D_14_) Mean ± SD	∆% vs. D_0_	Day 28 (D_28_) Mean ± SD	∆% vs. D_0_
2.5%_w/w_ AAO	Skin pliability/firmness (R_0_)	0.355 ± 0.024 mm	0.322 ± 0.014 mm	−9.0% *** ^$$$^	0.308 ± 0.028 mm	−13.0% *** ^$$$^
	Gross elasticity (R_2_)	0.602 ± 0.066%	0.676 ± 0.052%	14.0% *** ^$^	0.759 ± 0.071%	28.0% *** ^$$$^
Placebo	Skin pliability/firmness (R_0_)	0.308 ± 0.047 mm	0.342 ± 0.037 mm	8.0%	0.342 ± 0.037 mm	12.0%
	Gross elasticity (R_2_)	0.507 ± 0.044%	0.521 ± 0.041%	3.0%	0.526 ± 0.038%	4.0%

*t*-test vs. D_0_ *** *p* < 0.001; ANOVA vs. Placebo ^$^ *p* < 0.05, ^$$$^ *p* < 0.001.

**Table 2:** Skin hydration and film repairing activity (Corneometry and TEWL average value ± SD) before and after one application of 2.5%_w/w_ AAO or Placebo (short-term test) and during the 28-day treatment (Long-term test).

**Short-Term Test**						
	**Check-Up** 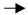 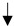 **Skin Parameter**	**Baseline** **Mean ± SD**	**After 1 h (1 h)** **Mean ± SD**	**∆% vs. Baseline**	**After 24 h (24 h)** **Mean ± SD**	**∆% vs. Baseline**
2.5%_w/w_ AAO	Skin conductance (Corneometry)	29.6 ± 1.8 A.U.	34.8 ± 5.9 A.U.	17.8% *** ^$^	34.6 ± 3.8 A.U.	17.2% *** ^$$^
	Trans epidermal water loss (TEWL)	9.0 ± 0.5 g/hm^2^	7.8 ± 1.1 g/hm^2^	−13.2% *** ^$$^	7.8 ± 1.1 g/hm^2^	−13.4% *** ^$$^
Placebo	Skin conductance (Corneometry)	32.0 ± 9.7 A.U.	33.3 ± 10.2 A.U.	4.8% ^NS^	32.0 ± 9.6 A.U.	1.3% ^NS^
	Trans epidermal water loss (TEWL)	7.7 ± 1.4 g/hm^2^	7.8 ± 1.3 g/hm^2^	3.5% ^NS^	7.9 ± 2.4 g/hm^2^	4.1% ^NS^
**Long-Term Test**						
	**Visit Day** 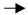 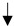 **Skin Parameter**	**Day 0 (D_0_)** **Mean ± SD**	**Day 14 (D_14_)** **Mean ± SD**	**∆% vs. D_0_**	**Day 28 (D_28_)** **Mean ± SD**	**∆% vs. D_0_**
2.5%_w/w_ AAO	Skin conductance (Corneometry)	29.6 ± 1.8 A.U.	35.6 ± 3.7 A.U.	20.0% *** ^$$$^	37.0 ± 3.2 A.U.	25.0% *** ^$$$^
	Trans epidermal water loss (TEWL)	9.0 ± 0.5 g/hm^2^	7.5 ± 0.8 g/hm^2^	−17.0% *** ^$$$^	6.9 ± 0.7 g/hm^2^	−23.0% *** ^$$$^
Placebo	Skin conductance (Corneometry)	31.0 ± 4.4 A.U.	31.9 ± 4.4 A.U.	3.1% ^NS^	32.3 ± 4.9 A.U.	4.3% ^NS^
	Trans epidermal water loss (TEWL)	7.7 ± 1.4 g/hm^2^	7.9 ± 1.1 g/hm^2^	5.0% ^NS^	8.2 ± 1.3 g/hm^2^	8.0% ^NS^

*t*-test vs. D_0_; *** *p* < 0.001; ANOVA vs. Placebo ^$^ *p* < 0.05, ^$$^ *p* < 0.01, ^$$$^ *p* < 0.001, ^NS^ *p* > 0.05, A.U. arbitrary units.

**Table 3:** Characteristics of clinical study participants.

Characteristics of Study Participants	2.5%_w/w_ AAO	Placebo
No. of subjects		
Female, *n* (%)	20 (100)	20 (100)
Male, *n* (%)	-	-
Mean age ± SD ^a^, years		
(min–max)	51.1 ± 10.5 (41–63)	50.7 ± 11.3 (40–64)
Fitzpatrick skin phototype, *n* (%)		
I	-	1 (5)
II	15 (75)	13 (65)
III	5 (25)	6 (30)
IV	-	-
Skin type, *n* (%)		
Dry	20 (100)	20 (100)
Normal	-	-
Combined	-	-
Oily	-	-

^a^ SD Standard deviation.

## Data Availability

The data used to support the findings of this study are included in this article.

## Supplementary Materials

The following supporting information can be downloaded at: https://www.mdpi.com/article/10.3390/ijms25031677/s1.

## References

[1] Cevenini E., Invidia L., Lescai F., Salvioli S., Tieri P., Castellani G., Franceschi C. (2008). Human Models of Aging and Longevity. Expert. Opin. Biol. Ther..

[2] Papaccio F., D’arino A., Caputo S., Bellei B. (2022). Focus on the Contribution of Oxidative Stress in Skin Aging. Antioxidants.

[3] Pérez-Sánchez A., Barrajón-Catalán E., Herranz-López M., Micol V. (2018). Nutraceuticals for Skin Care: A Comprehensive Review of Human Clinical Studies. Nutrients.

[4] Avola R., Graziano A.C.E., Pannuzzo G., Bonina F., Cardile V. (2019). Hydroxytyrosol from Olive Fruits Prevents Blue-Light-Induced Damage in Human Keratinocytes and Fibroblasts. J. Cell Physiol..

[5] Liu J.K. (2022). Natural Products in Cosmetics. Nat. Prod. Bioprospect..

[6] Feldo M., Wójciak M., Ziemlewska A., Dresler S., Sowa I. (2022). Modulatory Effect of Diosmin and Diosmetin on Metalloproteinase Activity and Inflammatory Mediators in Human Skin Fibroblasts Treated with Lipopolysaccharide. Molecules.

[7] Baylac S., Racine P. (2004). Inhibition of Human Leukocyte Elastase by Natural Fragrant Extracts of Aromatic Plants. Int. J. Aromather..

[8] Laneri S., Di Lorenzo R., Sacchi A., Dini I. (2019). Dosage of Bioactive Molecules in the Nutricosmeceutical Helix Aspersa Muller Mucus and Formulation of New Cosmetic Cream with Moisturizing Effect. Nat. Prod. Commun..

[9] Maisto M., Piccolo V., Novellino E., Schiano E., Iannuzzo F., Ciampaglia R., Summa V., Tenore G.C. (2023). Optimization of Ursolic Acid Extraction in Oil from Annurca Apple to Obtain Oleolytes with Potential Cosmeceutical Application. Antioxidants.

[10] Di Lorenzo R., Bernardi A., Grumetto L., Sacchi A., Avagliano C., Coppola S., Severina A.F. (2021). de G. di S.; Bruno, C.; Paparo, L.; Laneri, S.; et al. Phenylalanine Butyramide Is a New Cosmetic Ingredient with Soothing and Anti-Reddening Potential. Molecules.

[11] Perugini P., Vettor M., Rona C., Troisi L., Villanova L., Genta I., Conti B., Pavanetto F. (2008). Efficacy of Oleuropein against UVB Irradiation: Preliminary Evaluation. Int. J. Cosmet. Sci..

[12] Lim H.J., Kang S.H., Song Y.J., Jeon Y.D., Jin J.S. (2021). Inhibitory Effect of Quercetin on Propionibacterium Acnes-Induced Skin Inflammation. Int. Immunopharmacol..

[13] Amer S.S., Nasr M., Abdel-Aziz R.T.A., Moftah N.H., El Shaer A., Polycarpou E., Mamdouh W., Sammour O. (2020). Cosm-Nutraceutical Nanovesicles for Acne Treatment: Physicochemical Characterization and Exploratory Clinical Experimentation. Int. J. Pharm..

[14] Morganti P., Gao X., Vukovic N., Gagliardini A., Lohani A., Morganti G. (2022). Food Loss and Food Waste for Green Cosmetics and Medical Devices for a Cleaner Planet. Cosmetics.

[15] Ko K., Dadmohammadi Y., Abbaspourrad A. (2021). Nutritional and Bioactive Components of Pomegranate Waste Used in Food and Cosmetic Applications: A Review. Foods.

[16] Morganti P., Chen H.D., Gao X., Morganti G., Febo D. (2019). Chitin & Lignin: Turning Food Waste into Cosmeceuticals. J. Clin. Cosmet. Dermatol..

[17] Abbas S., Shanbhag T., Kothare A. (2021). Applications of Bromelain from Pineapple Waste towards Acne. Saudi J. Biol. Sci..

[18] Costa A., Marques M., Congiu F., Paiva A., Simões P., Ferreira A., Bronze M.R., Marto J., Ribeiro H.M., Simões S. (2021). Evaluating the Presence of Lycopene-Enriched Extracts from Tomato on Topical Emulsions: Physico-Chemical Characterization and Sensory Analysis. Appl. Sci..

[19] Do Nascimento P.G.G., Lemos T.L.G., Bizerra A.M.C., Arriaga A.M.C., Ferreira D.A., Santiago G.M.P., Braz-Filho R., Costa J.G.M. (2014). Antibacterial and Antioxidant Activities of Ursolic Acid and Derivatives. Molecules.

[20] Soleymani S., Farzaei M.H., Zargaran A., Niknam S., Rahimi R. (2020). Promising Plant-Derived Secondary Metabolites for Treatment of Acne Vulgaris: A Mechanistic Review. Arch. Dermatol. Res..

[21] Yo K., Oba A., Tada A. (2013). Novel Approach for Improving Skin Roughness Mediated by Ker-Atin Intermediate Filaments. J. Dermatol. Sci..

[22] Lim S.W., Hong S.P., Jeong S.W., Kim B., Bak H., Ryoo H.C., Lee S.H., Ahn S.K. (2007). Simultaneous Effect of Ursolic Acid and Oleanolic Acid on Epidermal Permeability Barrier Function and Epidermal Keratinocyte Differentiation via Peroxisome Proliferator-Activated Receptor-α. J. Dermatol..

[23] Neimkhum W., Anuchapreeda S., Lin W.C., Lue S.C., Lee K.H., Chaiyana W. (2021). Effects of Carissa Carandas Linn. Fruit, Pulp, Leaf, and Seed on Oxidation, Inflammation, Tyrosinase, Matrix Metalloproteinase, Elastase, and Hyaluronidase Inhibition. Antioxidants.

[24] López-Hortas L., Pérez-Larrán P., González-Muñoz M.J., Falqué E., Domínguez H. (2018). Recent Developments on the Extraction and Application of Ursolic Acid. A Review. Food Res. Int..

[25] Nkuimi Wandjou J.G., Lancioni L., Barbalace M.C., Hrelia S., Papa F., Sagratini G., Vittori S., Dall’Acqua S., Caprioli G., Beghelli D. (2020). Comprehensive Characterization of Phytochemicals and Biological Activities of the Italian Ancient Apple ‘Mela Rosa Dei Monti Sibillini’. Food Res. Int..

[26] Lo Scalzo R., Testoni A., Genna A. (2001). “Annurca” Apple Fruit, a Southern Italy Apple Cultivar: Textural Properties and Aroma Composition. Food Chem..

[27] Tenore G.C., Carotenuto A., Caruso D., Buonomo G., D’Avino M., Brancaccio D., Ciampaglia R., Maisto M., Schisano C., Novellino E. (2018). A Nutraceutical Formulation Based on Annurca Apple Polyphenolic Extract Is Effective on Intestinal Cholesterol Absorption: A Randomised, Placebo-Controlled, Crossover Study. PharmaNutrition.

[28] Maisto M., Schiano E., Novellino E., Piccolo V., Iannuzzo F., Salviati E., Summa V., Annunziata G., Tenore G.C. (2022). Application of a Rapid and Simple Technological Process to Increase Levels and Bioccessibility of Free Phenolic Compounds in Annurca Apple Nutraceutical Product. Foods.

[29] Maisto M., Piccolo V., Novellino E., Schiano E., Iannuzzo F., Ciampaglia R., Summa V., Tenore G.C. (2022). Optimization of Phlorizin Extraction from Annurca Apple Tree Leaves Using Response Surface Methodology. Antioxidants.

[30] Piccolo M., Ferraro M.G., Maione F., Maisto M., Stornaiuolo M., Tenore G.C., Santamaria R., Irace C., Novellino E. (2019). Induction of Hair Keratins Expression by an Annurca Apple-Based Nutraceutical Formulation in Human Follicular Cells. Nutrients.

[31] Nema N.K., Maity N., Sarkar B.K., Mukherjee P.K. (2013). Matrix Metalloproteinase, Hyaluronidase and Elastase Inhibitory Potential of Standardized Extract of Centella Asiatica. Pharm. Biol..

[32] Ambarwati N.S.S., Elya B., Desmiaty Y. (2019). Anti-Elastase Activity of Methanolic and Ethyl Acetate Extract from Garcinia Latissima Miq. J. Phys. Conf. Ser..

[33] Beatty M.W., Wee A.G., Marx D.B., Ridgway L., Simetich B., De Sousa T.C., Vakilzadian K., Schulte J. (2023). Viscoelastic Properties of Human Facial Skin and Comparisons with Facial Prosthetic Elastomers. Materials.

[34] Everett J.S., Sommers M.S. (2013). Skin Viscoelasticity: Physiologic Mechanisms, Measurement Issues, and Application to Nursing Science. Biol. Res. Nurs..

[35] Choi J.W., Kwon S.H., Huh C.H., Park K.C., Youn S.W. (2013). The Influences of Skin Visco-Elasticity, Hydration Level and Aging on the Formation of Wrinkles: A Comprehensive and Objective Approach. Ski. Res. Technol..

[36] Tsuji N., Moriwaki S., Suzuki Y., Takema Y., Imokawa G. (2001). The Role of Elastases Secreted by Fibroblasts in Wrinkle Formation: Implication Through Selective Inhibition of Elastase Activity¶. Photochem. Photobiol..

[37] Matsumoto T., Ikuta N., Mori M., Nagayama K. (2010). Mechanics of Wrinkle Formation: Micromechanical Analysis of Skin Deformation during Wrinkle Formation in Ultraviolet-Irradiated Mice. Ski. Res. Technol..

[38] Gilchrest B.A. (1996). A Review of Skin Ageing and Its Medical Therapy. Br. J. Dermatol..

[39] Komane B., Vermaak I., Kamatou G., Summers B., Viljoen A. (2017). The Topical Efficacy and Safety of Citrullus Lanatus Seed Oil: A Short-Term Clinical Assessment. S. Afr. J. Bot..

[40] Imokawa G., Ishida K. (2015). Biological Mechanisms Underlying the Ultraviolet Radiation-Induced Formation of Skin Wrinkling and Sagging I: Reduced Skin Elasticity, Highly Associated with Enhanced Dermal Elastase Activity, Triggers Wrinkling and Sagging. Int. J. Mol. Sci..

[41] Lee K.K., Cho J.J., Park E.J., Choi J.D. (2001). Anti-Elastase and Anti-Hyaluronidase of Phenolic Substance from Areca Catechu as a New Anti-Ageing Agent. Int. J. Cosmet. Sci..

[42] Khare R., Upmanyu N., Jha M. (2019). Exploring the Potential Effect of Methanolic Extract of Salvia Officinalis Against UV Exposed Skin Aging: In Vivo and In Vitro Model. Curr. Aging Sci..

[43] Sultana N. (2011). Clinically Useful Anticancer, Antitumor, and Antiwrinkle Agent, Ursolic Acid and Related Derivatives as Medicinally Important Natural Product. J. Enzyme Inhib. Med. Chem..

[44] Naeimifar A., Ahmad Nasrollahi S., Samadi A., Talari R., Sajad Ale-nabi S., Massoud Hossini A., Firooz A. (2020). Preparation and Evaluation of Anti-Wrinkle Cream Containing Saffron Extract and Avocado Oil. J. Cosmet. Dermatol..

[45] Farwick M., Köhler T., Schild J., Mentel M., Maczkiewitz U., Pagani V., Bonfigli A., Rigano L., Bureik D., Gauglitz G.G. (2014). Pentacyclic Triterpenes from Terminalia Arjuna Show Multiple Benefits on Aged and Dry Skin. Ski. Pharmacol. Physiol..

[46] Lee H.K., Nam G.W., Kim S.H., Lee S.H. (2006). Phytocomponents of Triterpenoids, Oleanolic Acid and Ursolic Acid, Regulated Differently the Processing of Epidermal Keratinocytes via PPAR-α Pathway. Exp. Dermatol..

[47] Debelle L., Alix A.J.P. (1999). The Structures of Elastins and Their Function. Biochimie.

[48] Both D.M., Goodtzova K., Yarosh D.B., Brown D.A., Sharma C., Deutsch J.M. (2023). Upcycling in the Context of Biotechnology-Based Solutions for Food Quality, Loss, and Consumer Perception. Curr. Opin. Biotechnol..

[49] Maisto M., Annunziata G., Schiano E., Piccolo V., Iannuzzo F., Santangelo R., Ciampaglia R., Tenore G.C., Novellino E., Grieco P. (2021). Potential Functional Snacks: Date Fruit Bars Supplemented by Different Species of *Lactobacillus* spp. Foods.

[50] (2015). Quality Management Systems. Requirements.

[51] Saunders J., Wainwright P. (2003). Risk, Helsinki 2000 and the Use of Placebo in Medical Research. Clin. Med..

[52] Renner G., Audebert F., Burfeindt J., Calvet B., Caratas-Perifan M., Leal M.E., Gorni R., Long A., Meredith E., O’Sullivan Ú. (2017). Cosmetics Europe Guidelines on the Management of Undesirable Effects and Reporting of Serious Undesirable Effects from Cosmetics in the European Union. Cosmetics.

[53] Lazzarini R., Duarte I., Ferreira A.L. (2013). Patch Tests. An. Bras. Dermatol..

