# Supplementary material for: Annurca Apple Oleolite as Functional Ingredient for the Formulation of Cosmetics with Skin-Antiaging Activity
Source: Int J Mol Sci. 2024 Jan 30;25(3):1677. doi: 10.3390/ijms25031677 (PMC10855134; doi:10.3390/ijms25031677)
Supplement: Supplementary file 1 [file ijms-25-01677-s001.zip › ijms-2797558-supplementary.pdf]

Trans epidermal water loss (TEWL) D0 - 1h<br>2,5% w/w AAO formulation				
**# Volontario**	**D0**	**1h**	**Δ(1h - D0)**	**Δ(1h -D0)%**
1	8,200	9,700	1,50	18,3
2	8,500	9,240	0,74	8,7
3	9,000	6,700	-2,30	-25,6
4	8,200	8,200	0,00	0,0
5	8,700	7,700	-1,00	-11,5
6	9,500	8,140	-1,36	-14,3
7	9,300	6,060	-3,24	-34,8
8	9,700	8,200	-1,50	-15,5
9	8,400	7,800	-0,60	-7,1
10	9,400	7,470	-1,93	-20,5
11	8,300	6,600	-1,70	-20,5
12	9,300	9,160	-0,14	-1,5
13	9,100	7,800	-1,30	-14,3
14	8,800	7,700	-1,10	-12,5
15	9,500	8,840	-0,66	-6,9
16	9,300	6,200	-3,10	-33,3
17	9,900	9,100	-0,80	-8,1
18	8,900	7,820	-1,08	-12,1
19	9,200	5,500	-3,70	-40,2
20	8,700	7,600	-1,10	-12,6
**Media**	9,0	7,8	-1,2	**-13,2**
**Std. Dev**	0,5	1,1	1,3	
N. Volontari	20	20	20	
t-test	4,386991471			
Gradi di Libertà	38			
p	8,81664E-05			
	***			

Trans epidermal water loss (TEWL) D0 - 24h<br>2,5% w/w AAO formulation				
**# Volontario**	**D0**	**24h**	**Δ(24h - D0)**	**Δ(24h -D0)%**
1	8,200	8,100	-0,10	-1,2
2	8,500	7,010	-1,49	-17,5
3	9,000	6,350	-2,65	-29,4
4	8,200	8,800	0,60	7,3
5	8,700	8,400	-0,30	-3,4
6	9,500	6,880	-2,62	-27,6
7	9,300	6,600	-2,70	-29,0
8	9,700	7,500	-2,20	-22,7
9	8,400	7,900	-0,50	-6,0
10	9,400	9,130	-0,27	-2,9
11	8,300	6,500	-1,80	-21,7
12	9,300	8,880	-0,42	-4,5
13	9,100	9,000	-0,10	-1,1
14	8,800	9,100	0,30	3,4
15	9,500	8,270	-1,23	-12,9
16	9,300	5,500	-3,80	-40,9
17	9,900	6,500	-3,40	-34,3
18	8,900	8,900	0,00	0,0
19	9,200	6,900	-2,30	-25,0
20	8,700	8,800	0,10	1,1
**Media**	9,0	7,8	-1,2	**-13,4**
**Std. Dev**	0,5	1,1	1,3	
N. Volontari	20	20	20	
t-test	4,473848333			
Gradi di Libertà	38			
p	6,7635E-05			
	***			

Trans epidermal water loss (TEWL) D0 - 1h<br>PLACEBO				
**# Volontario**	**D0**	**1h**	**Δ(1h - D0)**	**Δ(1h -D0)%**
1	8,700	7,300	-1,40	-16,1
2	7,300	7,000	-0,30	-4,1
3	4,200	6,800	2,60	61,9
4	8,500	7,100	-1,40	-16,5
5	9,000	9,100	0,10	1,1
6	8,200	10,200	2,00	24,4
7	8,700	9,000	0,30	3,4
8	6,100	7,100	1,00	16,4
9	8,900	7,300	-1,60	-18,0
10	7,000	6,300	-0,70	-10,0
11	9,900	8,800	-1,10	-11,1
12	7,600	7,700	0,10	1,3
13	7,600	8,100	0,50	6,6
14	7,900	10,700	2,80	35,4
15	5,900	6,400	0,50	8,5
16	7,700	8,100	0,40	5,2
17	9,800	8,400	-1,40	-14,3
18	6,300	5,900	-0,40	-6,3
19	8,000	7,900	-0,10	-1,3
20	6,300	6,500	0,20	3,2
**Media**	7,7	7,8	0,1	**3,5**
**Std. Dev**	1,4	1,3	1,3	
N. Volontari	20	20	20	
t-test	0,245490124			
Gradi di Libertà	38			
p	0,807397999			

Trans epidermal water loss (TEWL) D0 - 24h<br>PLACEBO				
**# Volontario**	**D0**	**24h**	**Δ(24h - D0)**	**Δ(24h -D0)%**
1	8,700	7,600	-1,10	-12,6
2	7,300	7,700	0,40	5,5
3	4,200	6,800	2,60	61,9
4	8,500	7,200	-1,30	-15,3
5	9,000	13,090	4,09	45,4
6	8,200	11,500	3,30	40,2
7	8,700	8,500	-0,20	-2,3
8	6,100	6,700	0,60	9,8
9	8,900	11,200	2,30	25,8
10	7,000	6,400	-0,60	-8,6
11	9,900	7,600	-2,30	-23,2
12	7,600	5,200	-2,40	-31,6
13	7,600	6,000	-1,60	-21,1
14	7,900	12,600	4,70	59,5
15	5,900	7,900	2,00	33,9
16	7,700	7,000	-0,70	-9,1
17	9,800	7,400	-2,40	-24,5
18	6,300	5,800	-0,50	-7,9
19	8,000	6,400	-1,60	-20,0
20	6,300	4,800	-1,50	-23,8
**Media**	7,7	7,9	0,2	**4,1**
**Std. Dev**	1,4	2,4	2,2	
N. Volontari	20	20	20	
t-test	0,306678287			
Gradi di Libertà	38			
p	0,760762476			

Trans epidermal water loss (TEWL) D0 - D14<br>2,5% w/w AAO formulation				
**# Volontario**	**D0**	**D14**	**Δ(TD14 - D0)**	**Δ(D14 -D0)%**
1	8,20	7,95	-0,25	-3,0
2	8,50	6,92	-1,58	-18,5
3	9,00	6,10	-2,90	-32,2
4	8,20	7,40	-0,80	-9,8
5	8,70	7,25	-1,45	-16,7
6	9,50	8,04	-1,46	-15,4
7	9,30	6,06	-3,24	-34,8
8	9,70	8,11	-1,59	-16,4
9	8,40	7,03	-1,37	-16,3
10	9,40	7,17	-2,23	-23,7
11	8,30	7,60	-0,70	-8,4
12	9,30	8,96	-0,34	-3,7
13	9,10	7,94	-1,16	-12,7
14	8,80	7,40	-1,40	-15,9
15	9,50	8,97	-0,53	-5,6
16	9,30	7,12	-2,18	-23,4
17	9,90	8,10	-1,80	-18,2
18	8,90	7,12	-1,78	-20,0
19	9,20	6,75	-2,45	-26,6
20	8,70	7,17	-1,53	-17,6
**Media**	9,0	7,5	-1,5	**-17,0**
**Std. Dev**	0,5	0,8	0,8	
N. Volontari	20	20	20	
t-test	7,416844756			
Gradi di Libertà	38			
p	6,75899E-09			
	***			

Trans epidermal water loss (TEWL) D0 - D28<br>2,5% w/w AAO formulation				
**# Volontario**	**D0**	**D28**	**Δ(T28 - D0)**	**Δ(D28 -D0)%**
1	8,20	7,01	-1,19	-14,5
2	8,50	6,01	-2,49	-29,3
3	9,00	5,95	-3,05	-33,9
4	8,20	7,15	-1,05	-12,8
5	8,70	7,04	-1,66	-19,1
6	9,50	6,61	-2,89	-30,4
7	9,30	5,92	-3,38	-36,3
8	9,70	7,13	-2,57	-26,5
9	8,40	7,00	-1,40	-16,7
10	9,40	6,95	-2,45	-26,1
11	8,30	6,12	-2,18	-26,3
12	9,30	8,58	-0,72	-7,7
13	9,10	7,49	-1,61	-17,7
14	8,80	7,10	-1,70	-19,3
15	9,50	8,28	-1,22	-12,8
16	9,30	6,54	-2,76	-29,7
17	9,90	7,50	-2,40	-24,2
18	8,90	6,91	-1,99	-22,4
19	9,20	6,29	-2,91	-31,6
20	8,70	6,80	-1,90	-21,8
**Media**	9,0	6,9	-2,1	**-23,0**
**Std. Dev**	0,5	0,7	0,7	
N. Volontari	20	20	20	
t-test	10,67330468			
Gradi di Libertà	38			
p	5,41229E-13			
	***			

Trans epidermal water loss (TEWL) D0 - D14<br>PLACEBO				
**# Volontario**	**D0**	**D14**	**Δ(TD14 - D0)**	**Δ(D14 -D0)%**
1	8,70	7,25	-1,45	-16,7
2	7,30	7,14	-0,16	-2,2
3	4,20	6,88	2,68	63,8
4	8,50	7,17	-1,33	-15,6
5	9,00	9,15	0,15	1,7
6	8,20	9,72	1,52	18,5
7	8,70	9,61	0,91	10,5
8	6,10	7,34	1,24	20,3
9	8,90	7,42	-1,48	-16,6
10	7,00	6,60	-0,40	-5,7
11	9,90	8,45	-1,45	-14,6
12	7,60	7,83	0,23	3,0
13	7,60	8,31	0,71	9,3
14	7,90	9,17	1,27	16,1
15	5,90	6,90	1,00	16,9
16	7,70	8,71	1,01	13,1
17	9,80	8,64	-1,16	-11,8
18	6,30	6,25	-0,05	-0,8
19	8,00	8,25	0,25	3,1
20	6,30	6,74	0,44	7,0
**Media**	7,7	7,9	0,2	**5,0**
**Std. Dev**	1,4	1,1	1,2	
N. Volontari	20	20	20	
t-test	0,498222884			
Gradi di Libertà	38			
p	0,621198431			

Trans epidermal water loss (TEWL) D0 - D28<br>PLACEBO				
**# Volontario**	**D0**	**D28**	**Δ(D28 - D0)**	**Δ(D28 -D0)%**
1	8,70	8,60	-0,10	-1,1
2	7,30	7,47	0,17	2,3
3	4,20	5,96	1,76	41,9
4	8,50	7,64	-0,86	-10,1
5	9,00	10,2	1,20	13,3
6	8,20	10,0	1,75	21,3
7	8,70	10,6	1,93	22,2
8	6,10	7,70	1,60	26,2
9	8,90	9,52	0,62	7,0
10	7,00	7,40	0,40	5,7
11	9,90	9,06	-0,84	-8,5
12	7,60	8,20	0,60	7,9
13	7,60	7,56	-0,04	-0,5
14	7,90	9,67	1,77	22,4
15	5,90	7,19	1,29	21,9
16	7,70	7,25	-0,45	-5,8
17	9,80	8,40	-1,40	-14,3
18	6,30	5,98	-0,32	-5,1
19	8,00	8,40	0,40	5,0
20	6,30	6,83	0,53	8,4
**Media**	7,7	8,2	0,5	**8,0**
**Std. Dev**	1,4	1,3	1,0	
N. Volontari	20	20	20	
t-test	1,151690056			
Gradi di Libertà	38			
p	0,256643193			

**Skin conductance (Corneometry) D0 - 1h**<br>2,5% w/w AAO formulation				
**# Volontario**	**D0**	**1h**	**Δ(1h - D0)**	**Δ(1h -D0)%**
1	28,600	32,900	4,30	15,0
2	29,100	39,300	10,20	35,1
3	28,590	31,780	3,19	11,2
4	29,960	43,300	13,34	44,5
5	29,400	32,930	3,53	12,0
6	29,900	27,430	-2,47	-8,3
7	28,800	27,980	-0,82	-2,8
8	28,900	35,120	6,22	21,5
9	27,640	39,610	11,97	43,3
10	27,900	32,260	4,36	15,6
11	29,020	28,930	-0,09	-0,3
12	29,310	33,090	3,78	12,9
13	28,570	50,970	22,40	78,4
14	28,810	33,190	4,38	15,2
15	34,000	37,120	3,12	9,2
16	28,200	31,940	3,74	13,3
17	30,500	39,820	9,32	30,6
18	29,050	31,120	2,07	7,1
19	33,820	39,920	6,10	18,0
20	32,800	27,560	-5,24	-16,0
**Media**	29,6	34,8	5,2	**17,8**
**Std. Dev**	1,8	5,9	6,1	
N. Volontari	20	20	20	
t-test	3,718503531			
Gradi di Libertà	38			
p	0,000644645			
	***			

**Skin conductance (Corneometry) D0 - 24h**<br>2,5% w/w AAO formulation				
**# Volontario**	**D0**	**24h**	**Δ(24h - D0)**	**Δ(24h -D0)%**
1	28,600	36,520	7,92	27,7
2	29,100	41,580	12,48	42,9
3	28,590	30,670	2,08	7,3
4	29,960	39,100	9,14	30,5
5	29,400	31,680	2,28	7,8
6	29,900	33,700	3,80	12,7
7	28,800	32,340	3,54	12,3
8	28,900	30,700	1,80	6,2
9	27,640	30,640	3,00	10,9
10	27,900	37,560	9,66	34,6
11	29,020	32,390	3,37	11,6
12	29,310	30,540	1,23	4,2
13	28,570	42,380	13,81	48,3
14	28,810	32,850	4,04	14,0
15	34,000	36,480	2,48	7,3
16	28,200	31,720	3,52	12,5
17	30,500	39,760	9,26	30,4
18	29,050	34,830	5,78	19,9
19	33,820	33,720	-0,10	-0,3
20	32,800	33,710	0,91	2,8
**Media**	29,6	34,6	5,0	**17,2**
**Std. Dev**	1,8	3,8	4,0	
N. Volontari	20	20	20	
t-test	5,364350292			
Gradi di Libertà	38			
p	4,23347E-06			
	***			

**Skin conductance (Corneometry) D0 - 1h**<br>PLACEBO				
**# Volontario**	**D0**	**1h**	**Δ(1h - D0)**	**Δ(1h -D0)%**
1	30,560	36,000	5,44	17,8
2	50,140	44,460	-5,68	-11,3
3	23,220	28,300	5,08	21,9
4	34,520	33,840	-0,68	-2,0
5	27,060	23,560	-3,50	-12,9
6	30,560	23,040	-7,52	-24,6
7	17,340	17,360	0,02	0,1
8	26,240	25,280	-0,96	-3,7
9	34,980	42,160	7,18	20,5
10	35,760	45,600	9,84	27,5
11	32,400	31,340	-1,06	-3,3
12	35,100	38,720	3,62	10,3
13	55,920	56,300	0,38	0,7
14	33,160	42,880	9,72	29,3
15	34,120	35,580	1,46	4,3
16	13,740	14,340	0,60	4,4
17	41,560	34,140	-7,42	-17,9
18	31,140	31,680	0,54	1,7
19	25,220	27,100	1,88	7,5
20	27,160	34,200	7,04	25,9
**Media**	32,0	33,3	1,3	**4,8**
**Std. Dev**	9,7	10,2	5,1	
N. Volontari	20	20	20	
t-test	0,412871208			
Gradi di Libertà	38			
p	0,68202139			

**Skin conductance (Corneometry) D0 - 24h**<br>PLACEBO				
**# Volontario**	**D0**	**24h**	**Δ(24h - D0)**	**Δ(24h -D0)%**
1	30,560	31,160	0,60	2,0
2	50,140	44,000	-6,14	-12,2
3	23,220	25,600	2,38	10,2
4	34,520	26,020	-8,50	-24,6
5	27,060	29,480	2,42	8,9
6	30,560	25,400	-5,16	-16,9
7	17,340	22,640	5,30	30,6
8	26,240	30,360	4,12	15,7
9	34,980	35,560	0,58	1,7
10	35,760	40,760	5,00	14,0
11	32,400	34,400	2,00	6,2
12	35,100	37,000	1,90	5,4
13	55,920	53,620	-2,30	-4,1
14	33,160	42,860	9,70	29,3
15	34,120	29,340	-4,78	-14,0
16	13,740	14,800	1,06	7,7
17	41,560	44,700	3,14	7,6
18	31,140	28,460	-2,68	-8,6
19	25,220	24,940	-0,28	-1,1
20	27,160	18,800	-8,36	-30,8
**Media**	32,0	32,0	0,0	**1,3**
**Std. Dev**	9,7	9,6	4,8	
N. Volontari	20	20	20	
t-test	0			
Gradi di Libertà	38			
p	1,000000			

Skin conductance (Corneometry) D0 - D14<br>2,5% w/w AAO formulation				
**# Volontario**	**D0**	**D14**	**Δ(D14 - D0)**	**Δ(D14 -D0)%**
1	28,60	31,50	2,90	10,1
2	29,10	36,23	7,13	24,5
3	28,59	32,83	4,24	14,8
4	29,96	43,17	13,21	44,1
5	29,40	34,29	4,89	16,6
6	29,90	30,53	0,63	2,1
7	28,80	29,90	1,10	3,8
8	28,90	36,21	7,31	25,3
9	27,64	38,46	10,82	39,1
10	27,90	35,13	7,23	25,9
11	29,02	31,19	2,17	7,5
12	29,31	32,95	3,64	12,4
13	28,57	37,90	9,33	32,6
14	28,81	35,62	6,81	23,6
15	34,00	39,12	5,12	15,1
16	28,20	33,59	5,39	19,1
17	30,50	41,38	10,88	35,7
18	29,05	33,56	4,51	15,5
19	33,82	40,58	6,76	20,0
20	32,80	36,86	4,06	12,4
**Media**	29,6	35,6	5,9	**20,0**
**Std. Dev**	1,8	3,7	3,3	
N. Volontari	20	20	20	
t-test	6,378166549			
Gradi di Libertà	38			
p	1,72646E-07			
	***			

Skin conductance (Corneometry) D0 - D28<br>2,5% w/w AAO formulation				
**# Volontario**	**D0**	**D28**	**Δ(D28 - D0)**	**Δ(D28 -D0)%**
1	28,60	35,72	7,12	24,9
2	29,10	40,54	11,44	39,3
3	28,59	34,67	6,08	21,3
4	29,96	40,91	10,95	36,5
5	29,40	36,56	7,16	24,4
6	29,90	35,17	5,27	17,6
7	28,80	34,53	5,73	19,9
8	28,90	35,97	7,07	24,5
9	27,64	39,42	11,78	42,6
10	27,90	37,14	9,24	33,1
11	29,02	34,09	5,07	17,5
12	29,31	33,14	3,83	13,1
13	28,57	41,32	12,75	44,6
14	28,81	36,28	7,47	25,9
15	34,00	40,34	6,34	18,6
16	28,20	30,89	2,69	9,5
17	30,50	39,26	8,76	28,7
18	29,05	35,98	6,93	23,9
19	33,82	43,17	9,35	27,6
20	32,80	35,10	2,30	7,0
**Media**	29,6	37,0	7,4	**25,0**
**Std. Dev**	1,8	3,2	2,9	
N. Volontari	20	20	20	
t-test	9,023535827			
Gradi di Libertà	38			
p	5,47722E-11			
	***			

Skin conductance (Corneometry) D0 - D14<br>PLACEBO					
**# Volontario**	**D0**	**D14**	**Δ(D14 - D0)**	**Δ(D14 -D0)%**	
1	30,56	32,00	1,44	4,7	
2	31,14	34,16	3,02	9,7	
3	23,22	25,43	2,21	9,5	
4	34,52	33,24	-1,28	-3,7	
5	27,06	26,28	-0,78	-2,9	
6	30,56	32,40	1,84	6,0	
7	27,34	29,03	1,69	6,2	
8	26,24	25,48	-0,76	-2,9	
9	34,98	32,61	-2,37	-6,8	
10	35,76	35,32	-0,44	-1,2	
11	32,40	33,13	0,73	2,3	
12	35,10	36,22	1,12	3,2	
13	30,92	31,59	0,67	2,2	
14	33,16	34,26	1,10	3,3	
15	34,12	35,81	1,69	5,0	
16	27,74	32,50	4,76	17,2	
17	31,56	32,00	0,44	1,4	1,4
18	31,14	33,88	2,74	8,8	
19	25,22	28,71	3,49	13,8	
20	27,16	23,62	-3,54	-13,0	
**Media**	30,5	31,4	0,9	**3,1**	
**Std. Dev**	3,6	3,7	2,0		
N. Volontari	20	20	20		
t-test	0,765852679				
Gradi di Libertà	38				
p	0,448495182				

Skin conductance (Corneometry) D0 - D28<br>PLACEBO				
**# Volontario**	**D0**	**D28**	**Δ(D28 - D0)**	**Δ(D28 -D0)%**
1	30,56	34,18	3,62	11,8
2	31,14	30,41	-0,73	-2,3
3	23,22	26,36	3,14	13,5
4	34,52	34,02	-0,50	-1,4
5	27,06	28,14	1,08	4,0
6	30,56	31,58	1,02	3,3
7	27,34	30,67	3,33	12,2
8	26,24	23,53	-2,71	-10,3
9	34,98	31,95	-3,03	-8,7
10	35,76	36,27	0,51	1,4
11	32,40	34,84	2,44	7,5
12	35,10	37,32	2,22	6,3
13	30,92	33,58	2,66	8,6
14	33,16	36,96	3,80	11,5
15	34,12	37,83	3,71	10,9
16	27,74	30,73	2,99	10,8
17	31,56	30,57	-0,99	-3,1
18	31,14	35,94	4,80	15,4
19	25,22	29,19	3,97	15,8
20	27,16	21,58	-5,58	-20,5
**Media**	30,5	31,8	1,3	**4,3**
**Std. Dev**	3,6	4,5	2,8	
N. Volontari	20	20	20	
t-test	0,997968214			
Gradi di Libertà	38			
p	0,324607538			

Forehead wrinkles Score D0 - D14<br>2,5% w/w AAO formulation				
**# Volontario**	**D0**	**D14**	**Δ(D14 - D0)**	**Δ(D14 -D0)%**
1	27,228	23,789	-3,44	-12,6
2	29,144	25,502	-3,64	-12,5
3	25,483	27,996	2,51	9,9
4	28,381	24,740	-3,64	-12,8
5	29,339	24,268	-5,07	-17,3
6	28,333	26,344	-1,99	-7,0
7	25,883	26,504	0,62	2,4
8	26,869	26,524	-0,34	-1,3
9	24,541	23,241	-1,30	-5,3
10	24,278	23,373	-0,90	-3,7
11	23,535	24,210	0,68	2,9
12	28,549	25,648	-2,90	-10,2
13	25,725	24,787	-0,94	-3,6
14	27,962	27,603	-0,36	-1,3
15	25,727	23,465	-2,26	-8,8
16	26,912	25,617	-1,29	-4,8
17	28,565	25,954	-2,61	-9,1
18	25,994	23,631	-2,36	-9,1
19	24,345	22,156	-2,19	-9,0
20	27,643	25,178	-2,47	-8,9
**Media**	26,7	25,0	-1,7	**-6,1**
**Std. Dev**	1,8	1,5	1,8	
N. Volontari	20	20	20	
t-test	3,245056881			
Gradi di Libertà	38			
p	0,002452147			
	**			

Forehead wrinkles Score D0 - D28<br>2,5% w/w AAO formulation				
**# Volontario**	**D0**	**D28**	**Δ(D28 - D0)**	**Δ(D28 -D0)%**
1	27,228	24,440	-2,79	-10,24
2	29,144	25,718	-3,43	-11,76
3	25,483	24,420	-1,06	-4,17
4	28,381	26,300	-2,08	-7,33
5	29,339	25,257	-4,08	-13,91
6	28,333	26,259	-2,07	-7,32
7	25,883	26,983	1,10	4,25
8	26,869	24,808	-2,06	-7,67
9	24,541	25,331	0,79	3,22
10	24,278	23,220	-1,06	-4,36
11	23,535	22,268	-1,27	-5,38
12	28,549	24,420	-4,13	-14,46
13	25,725	24,922	-0,80	-3,12
14	27,962	22,537	-5,43	-19,40
15	25,727	23,859	-1,87	-7,26
16	26,912	24,645	-2,27	-8,42
17	28,565	25,846	-2,72	-9,52
18	25,994	24,115	-1,88	-7,23
19	24,345	22,955	-1,39	-5,71
20	27,643	26,445	-1,20	-4,33
**Media**	26,7	24,7	-2,0	**-7,2**
**Std. Dev**	1,8	1,3	1,6	
N. Volunteers	20	20	20	
t-test	4,027932808			
Gradi di Libertà	38			
p	0,000259906			
	***			

Forehead wrinkles Score D0 - D14<br>PLACEBO				
**# Volontario**	**D0**	**D14**	**Δ(D14 - D0)**	**Δ(D14 -D0)%**
1	26,300	28,442	2,14	8,1
2	27,611	25,502	-2,11	-7,6
3	35,359	40,345	4,99	14,1
4	37,454	40,488	3,03	8,1
5	29,340	30,481	1,14	3,9
6	26,747	28,344	1,60	6,0
7	34,977	35,804	0,83	2,4
8	36,223	35,786	-0,44	-1,2
9	31,120	41,153	10,03	32,2
10	28,807	29,738	0,93	3,2
11	30,992	34,473	3,48	11,2
12	30,577	33,196	2,62	8,6
13	27,614	25,198	-2,42	-8,7
14	34,826	35,404	0,58	1,7
15	28,157	30,984	2,83	10,0
16	32,494	35,166	2,67	8,2
17	30,666	34,811	4,15	13,5
18	29,157	30,444	1,29	4,4
19	32,904	36,961	4,06	12,3
20	33,619	34,538	0,92	2,7
**Media**	31,2	33,4	2,1	**6,7**
**Std. Dev**	3,3	4,6	2,7	
N. Volontari	20	20	20	
t-test	1,652370547			
Gradi di Libertà	38			
p	0,106699487			

Forehead wrinkles Score D0 - D28<br>PLACEBO				
**# Volontario**	**D0**	**D28**	**Δ(D28 - D0)**	**Δ(D28 -D0)%**
1	26,300	33,354	7,05	26,82
2	27,611	30,282	2,67	9,67
3	35,359	38,640	3,28	9,28
4	37,454	39,402	1,95	5,20
5	29,340	31,696	2,36	8,03
6	26,747	30,814	4,07	15,21
7	34,977	37,930	2,95	8,44
8	36,223	33,049	-3,17	-8,76
9	31,120	30,002	-1,12	-3,59
10	28,807	29,988	1,18	4,10
11	30,992	37,173	6,18	19,94
12	30,577	28,942	-1,64	-5,35
13	27,614	28,776	1,16	4,21
14	34,826	37,017	2,19	6,29
15	28,157	35,000	6,84	24,30
16	32,494	30,068	-2,43	-7,47
17	30,666	36,306	5,64	18,39
18	29,157	33,867	4,71	16,15
19	32,904	38,571	5,67	17,22
20	33,619	34,638	1,02	3,03
**Media**	31,2	33,8	2,5	**8,6**
**Std. Dev**	3,3	3,6	3,0	
N. Volunteers	20	20	20	
t-test	2,309887886			
Gradi di Libertà	38			
p	0,026421785			

**Nasolabial wrinkles Score D0 - D14**<br>2,5% w/w AAO formulation				
**# Volontario**	**D0**	**D14**	**Δ(D14 - D0)**	**Δ(D14 -D0)%**
1	55,425	56,866	1,44	2,6
2	55,953	58,261	2,31	4,1
3	54,684	50,567	-4,12	-7,5
4	56,974	52,473	-4,50	-7,9
5	54,714	57,960	3,25	5,9
6	60,594	56,170	-4,42	-7,3
7	60,122	54,771	-5,35	-8,9
8	54,674	53,580	-1,09	-2,0
9	59,624	58,770	-0,85	-1,4
10	57,913	50,789	-7,12	-12,3
11	58,270	56,942	-1,33	-2,3
12	58,467	54,183	-4,28	-7,3
13	52,700	58,233	5,53	10,5
14	53,286	52,024	-1,26	-2,4
15	56,284	55,188	-1,10	-1,9
16	51,947	52,184	0,24	0,5
17	61,163	55,623	-5,54	-9,1
18	58,911	56,382	-2,53	-4,3
19	60,182	58,436	-1,75	-2,9
20	55,117	51,861	-3,26	-5,9
**Media**	56,9	55,1	-1,8	**-3,0**
**Std. Dev**	2,8	2,7	3,2	
N. Volontari	20	20	20	
t-test	2,05941889			
Gradi di Libertà	38			
p	0,046353681			
	*			

**Nasolabial wrinkles Score D0 - D28**<br>2,5% w/w AAO formulation				
**# Volontario**	**D0**	**D28**	**Δ(D28 - D0)**	**Δ(D28 -D0)%**
1	55,425	54,982	-0,44	-0,80
2	55,953	53,893	-2,06	-3,68
3	54,684	48,889	-5,79	-10,60
4	56,974	53,070	-3,90	-6,85
5	54,714	50,604	-4,11	-7,51
6	60,594	59,491	-1,10	-1,82
7	60,122	57,374	-2,75	-4,57
8	54,674	47,953	-6,72	-12,29
9	59,624	56,327	-3,30	-5,53
10	57,913	51,571	-6,34	-10,95
11	58,270	59,090	0,82	1,41
12	58,467	53,858	-4,61	-7,88
13	52,700	55,790	3,09	5,86
14	53,286	54,891	1,61	3,01
15	56,284	52,932	-3,35	-5,96
16	51,947	49,783	-2,16	-4,17
17	61,163	57,964	-3,20	-5,23
18	58,911	53,762	-5,15	-8,74
19	60,182	55,888	-4,29	-7,14
20	55,117	51,584	-3,53	-6,41
**Media**	56,9	54,0	-2,9	**-5,0**
**Std. Dev**	2,8	3,3	2,6	
N. Volunteers	20	20	20	
t-test	2,989191253			
Gradi di Libertà	38			
p	0,004883921			
	**			

**Nasolabial wrinkles Score D0 - D14**<br>PLACEBO				
**# Volontario**	**D0**	**D14**	**Δ(D14 - D0)**	**Δ(D14 -D0)%**
1	60,126	63,398	3,27	5,4
2	53,586	56,784	3,20	6,0
3	56,665	57,194	0,53	0,9
4	62,320	64,910	2,59	4,2
5	52,675	56,368	3,69	7,0
6	55,827	55,830	0,00	0,0
7	49,146	51,954	2,81	5,7
8	56,561	50,030	-6,53	-11,5
9	48,999	54,321	5,32	10,9
10	56,548	58,762	2,21	3,9
11	69,888	79,057	9,17	13,1
12	51,505	53,530	2,03	3,9
13	54,797	58,666	3,87	7,1
14	49,849	53,414	3,57	7,2
15	48,496	48,266	-0,23	-0,5
16	57,983	60,592	2,61	4,5
17	61,309	62,927	1,62	2,6
18	60,881	61,930	1,05	1,7
19	47,978	49,639	1,66	3,5
20	49,579	51,641	2,06	4,2
**Media**	55,2	57,5	2,2	**4,0**
**Std. Dev**	5,8	7,0	2,9	
N. Volontari	20	20	20	
t-test	1,097812301			
Gradi di Libertà	38			
p	0,279194217			

**Nasolabial wrinkles Score D0 - D28**<br>PLACEBO				
**# Volontario**	**D0**	**D28**	**Δ(D28 - D0)**	**Δ(D28 -D0)%**
1	60,126	65,992	5,87	9,76
2	53,586	59,662	6,08	11,34
3	56,665	58,770	2,11	3,71
4	62,320	68,839	6,52	10,46
5	52,675	58,683	6,01	11,41
6	55,827	58,907	3,08	5,52
7	49,146	51,225	2,08	4,23
8	56,561	53,995	-2,57	-4,54
9	48,999	52,548	3,55	7,24
10	56,548	56,735	0,19	0,33
11	69,888	79,982	10,09	14,44
12	51,505	53,752	2,25	4,36
13	54,797	56,910	2,11	3,86
14	49,849	59,179	9,33	18,72
15	48,496	49,007	0,51	1,05
16	57,983	60,767	2,78	4,80
17	61,309	62,172	0,86	1,41
18	60,881	66,905	6,02	9,89
19	47,978	47,910	-0,07	-0,14
20	49,579	50,880	1,30	2,62
**Media**	55,2	58,6	3,4	**6,0**
**Std. Dev**	5,8	7,7	3,2	
N. Volunteers	20	20	20	
t-test	1,590333731			
Gradi di Libertà	38			
p	0,120045882			

Skin pliability/firmness (R0) D0 - D14
2,5% w/w AAO formulation

# Volontario	D0	D14	Δ(D14 - D0)	Δ(D14 -D0)%
1	0,296	0,291	-0,01	-1,7
2	0,365	0,331	-0,03	-9,3
3	0,365	0,297	-0,07	-18,6
4	0,334	0,313	-0,02	-6,3
5	0,387	0,343	-0,04	-11,4
6	0,333	0,318	-0,01	-4,4
7	0,354	0,336	-0,02	-5,1
8	0,317	0,309	-0,01	-2,5
9	0,327	0,312	-0,02	-4,6
10	0,376	0,329	-0,05	-12,5
11	0,373	0,327	-0,05	-12,3
12	0,361	0,341	-0,02	-5,5
13	0,347	0,328	-0,02	-5,4
14	0,381	0,318	-0,06	-16,5
15	0,344	0,327	-0,02	-4,9
16	0,368	0,329	-0,04	-10,6
17	0,370	0,331	-0,04	-10,5
18	0,358	0,315	-0,04	-12,0
19	0,392	0,332	-0,06	-15,3
20	0,347	0,308	-0,04	-11,2
**Media**	0,355	0,322	0,0	**-9,0**
**Std. Dev**	0,024	0,014		
N. Volontari	20	20		
t-test	5,248439051			
Gradi di Libertà	38			
p	6,0956E-06			
	***			

Skin pliability/firmness (R0) D0 - D28
2,5% w/w AAO formulation

# Volontario	D0	D28	Δ(D28 - D0)	Δ(D28 -T0)%
1	0,296	0,296	0,00	0,0
2	0,365	0,303	-0,06	-17,0
3	0,365	0,294	-0,07	-19,5
4	0,334	0,293	-0,04	-12,3
5	0,387	0,303	-0,08	-21,7
6	0,333	0,293	-0,04	-12,0
7	0,354	0,346	-0,01	-2,3
8	0,317	0,280	-0,04	-11,7
9	0,327	0,295	-0,03	-9,8
10	0,376	0,310	-0,07	-17,6
11	0,373	0,370	0,00	-0,8
12	0,361	0,298	-0,06	-17,5
13	0,347	0,381	0,03	9,8
14	0,381	0,298	-0,08	-21,8
15	0,344	0,297	-0,05	-13,7
16	0,368	0,291	-0,08	-20,9
17	0,370	0,332	-0,04	-10,3
18	0,358	0,280	-0,08	-21,8
19	0,392	0,302	-0,09	-23,0
20	0,347	0,292	-0,06	-15,9
**Media**	0,355	0,308	-0,047	**-13,0**
**Std. Dev**	0,024	0,028		
N. Volontari	20	20	20	
t-test	5,684794358			
Gradi di Libertà	38			
p	1,54092E-06			
	***			

Skin pliability/firmness (R0) D0 - D14
PLACEBO

# Volontario	D0	D14	Δ(D14 - D0)	Δ(D14 -D0)%
1	0,287	0,323	0,04	12,5
2	0,332	0,343	0,01	3,3
3	0,359	0,313	-0,05	-12,8
4	0,371	0,384	0,01	3,5
5	0,250	0,258	0,01	3,2
6	0,341	0,352	0,01	3,2
7	0,368	0,391	0,02	6,3
8	0,385	0,370	-0,02	-3,9
9	0,301	0,398	0,10	32,2
10	0,277	0,316	0,04	14,1
11	0,285	0,335	0,05	17,5
12	0,348	0,341	-0,01	-2,0
13	0,303	0,379	0,08	25,1
14	0,281	0,293	0,01	4,3
15	0,235	0,306	0,07	30,2
16	0,349	0,341	-0,01	-2,3
17	0,317	0,327	0,01	3,2
18	0,227	0,234	0,01	3,1
19	0,301	0,356	0,06	18,3
20	0,248	0,251	0,00	1,2
**Media**	0,308	0,331	0,0	**8,0**
**Std. Dev**	0,047	0,046		
N. Volontari	20	20		
t-test	1,511579593			
Gradi di Libertà	38			
p	0,138913204			

Skin pliability/firmness (R0) D0 - D28
PLACEBO

# Volontario	D0	D28	Δ(D28 - D0)	Δ(D28 -T0)%
1	0,287	0,334	0,05	16,4
2	0,332	0,354	0,02	6,6
3	0,359	0,365	0,01	1,7
4	0,371	0,373	0,00	0,5
5	0,250	0,295	0,05	18,0
6	0,341	0,374	0,03	9,7
7	0,368	0,399	0,03	8,4
8	0,385	0,372	-0,01	-3,4
9	0,301	0,368	0,07	22,3
10	0,277	0,324	0,05	16,8
11	0,285	0,315	0,03	10,5
12	0,348	0,354	0,01	1,7
13	0,303	0,337	0,03	11,2
14	0,281	0,323	0,04	14,9
15	0,235	0,336	0,10	43,0
16	0,349	0,368	0,02	5,4
17	0,317	0,365	0,05	15,1
18	0,227	0,258	0,03	13,7
19	0,301	0,365	0,06	21,3
20	0,248	0,264	0,02	6,5
**Media**	0,308	0,342	0,0	**12,0**
**Std. Dev**	0,047	0,037		
N. Volontari	20	20	20	
t-test	2,514463308			
Gradi di Libertà	38			
p	0,016274168			

Gross Elasticity (R2) D0 - D14<br>2,5% w/w AAO formulation				
# Volontario	D0	D14	Δ(D14 - D0)	Δ(D14 -D0)%
1	0,487	0,752	0,27	54,4
2	0,497	0,716	0,22	44,1
3	0,596	0,654	0,06	9,7
4	0,604	0,612	0,01	1,3
5	0,598	0,705	0,11	17,9
6	0,587	0,606	0,02	3,2
7	0,694	0,698	0,00	0,6
8	0,506	0,699	0,19	38,1
9	0,696	0,742	0,05	6,6
10	0,676	0,741	0,06	9,6
11	0,607	0,689	0,08	13,5
12	0,519	0,629	0,11	21,2
13	0,695	0,598	-0,10	-14,0
14	0,694	0,615	-0,08	-11,4
15	0,591	0,706	0,12	19,5
16	0,597	0,711	0,11	19,1
17	0,603	0,719	0,12	19,2
18	0,571	0,703	0,13	23,1
19	0,592	0,633	0,04	6,9
20	0,623	0,602	-0,02	-3,4
**Media**	0,602	0,676	0,1	**14,0**
**Std. Dev**	0,066	0,052		
N. Volontari	20	20	20	
t-test	3,98870734			
Gradi di Libertà	38			
p	0,000292009			
	***			

Gross Elasticity (R2) D0 - D28<br>2,5% w/w AAO formulation				
# Volontario	D0	D28	Δ(D28 - D0)	Δ(D28 -D0)%
1	0,487	0,802	0,32	64,7
2	0,497	0,811	0,31	63,2
3	0,596	0,804	0,21	34,9
4	0,604	0,812	0,21	34,4
5	0,598	0,725	0,13	21,2
6	0,587	0,791	0,20	34,8
7	0,694	0,698	0,00	0,6
8	0,506	0,759	0,25	50,0
9	0,696	0,842	0,15	21,0
10	0,676	0,831	0,16	22,9
11	0,607	0,609	0,00	0,3
12	0,519	0,789	0,27	52,0
13	0,695	0,691	0,00	-0,6
14	0,694	0,646	-0,05	-6,9
15	0,591	0,818	0,23	38,4
16	0,597	0,812	0,22	36,0
17	0,603	0,861	0,26	42,8
18	0,571	0,703	0,13	23,1
19	0,592	0,683	0,09	15,4
20	0,623	0,702	0,08	12,7
**Media**	0,602	0,759	0,2	**28,0**
**Std. Dev**	0,066	0,071		
N. Volontari	20	20	20	
t-test	7,266434544			
Gradi di Libertà	38			
p	1,0754E-08			
	***			

Gross Elasticity (R2) D0 - D14<br>PLACEBO				
# Volontario	D0	D14	Δ(D14 - D0)	Δ(D14 -D0)%
1	0,512	0,528	0,02	3,1
2	0,538	0,557	0,02	3,5
3	0,593	0,591	0,00	-0,3
4	0,425	0,504	0,08	18,6
5	0,475	0,496	0,02	4,4
6	0,512	0,529	0,02	3,3
7	0,498	0,425	-0,07	-14,7
8	0,482	0,506	0,02	4,9
9	0,512	0,508	0,00	-0,8
10	0,498	0,511	0,01	2,6
11	0,434	0,451	0,02	3,9
12	0,467	0,464	0,00	-0,6
13	0,569	0,562	-0,01	-1,2
14	0,543	0,546	0,00	0,6
15	0,547	0,551	0,00	0,7
16	0,502	0,514	0,01	2,4
17	0,537	0,551	0,01	2,6
18	0,531	0,542	0,01	2,1
19	0,527	0,566	0,04	7,4
20	0,437	0,512	0,08	17,2
**Media**	0,507	0,521	0,0	**3,0**
**Std. Dev**	0,044	0,041		
N. Volontari	20	20	20	
t-test	1,01977079			
Gradi di Libertà	38			
p	0,314286017			

Gross Elasticity (R2) D0 - D28<br>PLACEBO				
# Volontario	D0	D28	Δ(D28 - D0)	Δ(D28 -D0)%
1	0,512	0,521	0,01	1,8
2	0,538	0,551	0,01	2,4
3	0,593	0,574	-0,02	-3,2
4	0,425	0,512	0,09	20,5
5	0,475	0,425	-0,05	-10,5
6	0,512	0,561	0,05	9,6
7	0,498	0,521	0,02	4,6
8	0,482	0,495	0,01	2,7
9	0,512	0,542	0,03	5,9
10	0,498	0,517	0,02	3,8
11	0,434	0,469	0,04	8,1
12	0,467	0,489	0,02	4,7
13	0,569	0,561	-0,01	-1,4
14	0,543	0,546	0,00	0,6
15	0,547	0,584	0,04	6,7
16	0,502	0,512	0,01	2,0
17	0,537	0,561	0,02	4,5
18	0,531	0,533	0,00	0,4
19	0,527	0,531	0,00	0,8
20	0,437	0,506	0,07	15,8
**Media**	0,507	0,526	0,0	**4,0**
**Std. Dev**	0,044	0,038		
N. Volontari	20	20	20	
t-test	1,424323801			
Gradi di Libertà	38			
p	0,162513647			

#	Panelist ID	Sex	Erythema 48h	Oedema 48	Erythema 72h	Oedema 72h	
1	FF85	F	0	0	0	0	
2	LR95	F	0	0	0	0	
3	FDL76	F	0	1	0	0	
4	RDL62	F	2	0	0	0	
5	EV79	F	0	0	0	0	
6	FM68	F	0	0	0	0	
7	AM68	F	1	0	0	0	
8	BA74	F	1	1	0	0	
9	IN77	F	1	2	0	1	
10	VN76	F	0	0	0	0	
11	SN78	F	0	0	0	0	
12	FS77	F	1	1	0	0	
13	IL66	F	0	0	0	0	
14	AD66	F	0	0	0	0	
15	PR71	F	0	0	0	0	
16	AG66	F	0	1	0	0	
17	LR88	F	0	0	0	0	
18	MDC76	F	0	0	0	0	
19	LB77	F	0	0	0	0	
20	EF66	F	0	0	0	0	
MEAN VALUES			**0,300**	**0,300**	**0,000**	**0,050**	**0,050**
SD			0,571	0,571	0,000	0,224	
MEAN + SD			**0,871**	**0,871**	**0,000**	**0,274**	**0,274**
			E-PI48	*O-PI48*	*E-PI72*	*O-PI72*	*PIM*

UNIVERSITÀ DEGLI STUDI DI NAPOLI FEDERICO II

PATIENT CONSENT FORM

Name.. Surname ....

Study Title: .... Type of study..

a) **PREDICTABLE RISKS LINKED TO EXECUTION (AT THE TIME OF PROPOSAL) AND POSSIBLE COMPLICATIONS:** redness (erythema) may appear in the treated area during the study, which usually regresses in 24-48 hours; blisters and/or scabs may appear and disappear in 10-20 days; soothing, healing and disinfectant dressings are prescribed for both situations. In some cases, localized and/or extensive sensitivity of treated areas may occur. In case of severe irritation, hives, swelling of the eyes and mouth, blisters, rinse, discontinue use and seek immediate medical attention.

b) **ANY PRECIFICATIONS DUE TO PARTICULAR CLINICAL CONDITIONS:** in the case of subjects with ongoing infectious or widespread inflammatory processes, subjects undergoing therapy with photosensitizing drugs, and subjects with a positive history of hypertrophic or keloid scars, allergic subjects, subjects with rosacea, psoriasis, atopic dermatitis, or other dermatological conditions, subjects undergoing treatment with AHAs and BHAs, retinoids or derivatives, whether topical or systemic, cosmetic treatment is not recommended.

Dipartimento di Farmacia
Via Domenico Montesano, 49 • 80131 Napoli, Italia
slaneri@unina.it • + 39 081678650
www.farmacia.unina.it

I, the undersigned ....document of identification .... no. ....

On ..../......./.......... now .... during the interview with Dr./Prof. .... I was informed about the proposal to be subjected to:

COSMETIC EFFICACY STUDY REF. **FBA CEE 21N01**

I have been given the explanations listed above under a) b) and having understood what was explained, I freely and knowingly decide to:

☐ Consent to be subjected to the above-described service, taking into account also the foreseeable impairing consequences, temporary or permanent, resulting from the performance of the proposed service, as described in the Disclosure.

I acknowledge that I have received and read a copy of the Specific Written Notice.
I understand that it is my right to request further explanation at any time.
I am also aware that I may revoke my decisions expressed herein at any time.

- ✓ **I commit myself, to take part in the product efficacy study for the duration of the product test product ....**
- ✓ **I also agree to comply with the protocol drafted by the investigators, specified in point a of the RD Cosmetics laboratory of the Department of Pharmacy - University of Naples Federico II, located in Via D. Montesano, 49 - 80131 Naples.**
- ✓ **I also commit myself not to use any other type of product on the skin, not previously agreed and accepted by the investigators, throughout the study period.**
- ✓ **I undertake to take part in the periodic controls established with the laboratory technical staff.**
- ✓ **Furthermore, having read the composition of the product (Id Form IOP04_M Sample Acceptance Form), I declare that I do not have any type of allergy to the components in it.**
- ✓ **I also declare that I have no allergies towards ingredients commonly used in cosmetic products.**
- ✓ **I am aware that all product safety data have been evaluated in accordance with Annex 1 of EC Regulation 1223/2009.**

I, the undersigned, further declare that I am aware in giving this consent and assume full responsibility for it.

Signature of Volunteer ....

Signature of Witness .... (Person present at the interview)

Stamp and Signature of Laboratory Manager ....

Dipartimento di Farmacia
Via Domenico Montesano, 49 • 80131 Napoli, Italia
slaneri@unina.it • + 39 081678650
www.farmacia.unina.it
